# α_2_-Macroglobulin-like protein 1 can conjugate and inhibit proteases through their hydroxyl groups, because of an enhanced reactivity of its thiol ester

**DOI:** 10.1074/jbc.RA120.015694

**Published:** 2021-01-13

**Authors:** Seandean Lykke Harwood, Nadia Sukusu Nielsen, Kathrine Tejlgård Jensen, Peter Kresten Nielsen, Ida B. Thøgersen, Jan J. Enghild

**Affiliations:** 1 Department of Molecular Biology and Genetics, Aarhus University, Aarhus, Denmark; 2 General Research Technologies, Novo Nordisk A/S, Måløv, Denmark

**Keywords:** alpha 2 macroglobulin like protein 1, A2ML1, α2-macroglobulin, thiol ester, complement, inhibition mechanism, protein crosslinking, mutagenesis in vitro, protease inhibitor, mutagenesis, protease, alpha-2-macroglobulin

## Abstract

Proteins in the α-macroglobulin (αM) superfamily use thiol esters to form covalent conjugation products upon their proteolytic activation. αM protease inhibitors use theirs to conjugate proteases and preferentially react with primary amines (*e.g.* on lysine side chains), whereas those of αM complement components C3 and C4B have an increased hydroxyl reactivity that is conveyed by a conserved histidine residue and allows conjugation to cell surface glycans. Human α_2_-macroglobulin–like protein 1 (A2ML1) is a monomeric protease inhibitor but has the hydroxyl reactivity–conveying histidine residue. Here, we have investigated the role of hydroxyl reactivity in a protease inhibitor by comparing recombinant WT A2ML1 and the A2ML1 H1084N mutant in which this histidine is removed. Both of A2ML1s' thiol esters were reactive toward the amine substrate glycine, but only WT A2ML1 reacted with the hydroxyl substrate glycerol, demonstrating that His-1084 increases the hydroxyl reactivity of A2ML1's thiol ester. Although both A2ML1s conjugated and inhibited thermolysin, His-1084 was required for the conjugation and inhibition of acetylated thermolysin, which lacks primary amines. Using MS, we identified an ester bond formed between a thermolysin serine residue and the A2ML1 thiol ester. These results demonstrate that a histidine-enhanced hydroxyl reactivity can contribute to protease inhibition by an αM protein. His-1084 did not improve A2ML1's protease inhibition at pH 5, indicating that A2ML1's hydroxyl reactivity is not an adaption to its acidic epidermal environment.

Human A2ML1 is a member of the αM protein superfamily of protease inhibitors and complement factors. It has been found in the epidermal granular layer of skin in intracellular keratinosomes and extracellularly in proximity to desmosomal interfaces between keratinocytes and has been shown to inhibit several proteases *in vitro* (1). It has since been implicated in the pathogenesis of several diseases, including paraneoplastic pemphigus (2) and otitis media (3, 4). Although it is suspected of regulating epidermal proteases, *e.g.* kallikreins involved in keratinocyte differentiation and desquamation (5, 6), its physiological role has not yet been determined.

Protease inhibitors of the αM protein superfamily possess a unique trapping mechanism whereby they pseudo-inhibit proteases (7). Human α2-macroglobulin (A2M) is the most extensively characterized αM protease inhibitor, and its study has identified several broadly applicable mechanistic steps (8). The inhibitory mechanism is initiated after cleavage by a protease in an especially susceptible sequence stretch called the bait region, which is largely unstructured and poorly conserved across different αM protease inhibitors or the same protease inhibitor in different species (9). When the bait region is cleaved, it triggers a drastic conformational change in the protease inhibitor, which collapses around the protease (10). Consequentially, a reactive thiol ester moiety moves from a protected and inaccessible position to a position where it is exposed to nucleophilic attack (11, 12). Reaction of the thiol ester with nucleophiles on the protease's surface, most commonly the ε-amino groups of lysine side chains, results in amide bond formation that covalently traps the protease (13, 14). αM protease inhibitors can be found as functional monomers, as is the case for A2ML1 (1), rat α_1_-I_3_ (15), and *Escherichia coli* ECAM (16), dimers such as human pregnancy zone protein (PZP) (17) and *Limulus* α-macroglobulin complement-like protein (LIMAC) (18), and tetramers such as A2M and ovostatins (19). The addition of a surplus of competing nucleophiles, *e.g.* 3-aminopropanenitrile (BAPN), during protease inhibition prevents covalent protease trapping; whereas rat α_1_-I_3_, ECAM, and human PZP require covalent trapping to inhibit proteases (15, 16, 17), noncovalent binding is sufficient for αM tetramers (20).

αM complement factors also undergo proteolytically induced conformational changes, but rather than trapping the convertases by which they are cleaved, they instead use their thiol ester to conjugate to cell surfaces. The thiol esters of complement components C3 and C4B have an increased reactivity toward hydroxyl groups compared with A2M (21) that enables them to conjugate glycans. The source of this altered reactivity has been identified as a conserved histidine residue located in close proximity to the thiol ester, which reacts with the thiol ester to form an acyl-imidazole intermediate after the complement factor is proteolytically activated (22). The acyl-imidazole intermediate is reactive and short-lived and reacts with the most abundant hydroxyl, water, with a *t*_1/2_ of less than a second (23).

Human A2ML1, in contrast to other known αM protease inhibitors (16, 21, 24), has a histidine residue at position 1084, which is equivalent to the position of the acyl-imidazole–forming histidine residues of C3 and C4B. To investigate the role of A2ML1's potential hydroxyl reactivity in protease inhibition, we have compared WT A2ML1 with the A2ML1 H1084N mutant, which lacks the acyl-imidazole–forming histidine. His-1084 conveys hydroxyl reactivity in A2ML1 and is required for the thiol ester to conjugate the hydroxyl substrate glycerol after A2ML1 is proteolytically activated. In assays using both unmodified and acetylated thermolysin, we have shown that this hydroxyl reactivity allows WT A2ML1 to conjugate proteases through their hydroxyl groups. This expands the inhibitory repertoire of A2ML1 to include proteases without accessible amine groups but does not compromise its inhibition of proteases with normal surface lysine densities, demonstrating a functional role for hydroxyl reactivity in A2ML1 and possibly other protease inhibitors.

## Results

### His-1084 conveys an increased reactivity of the A2ML1 thiol ester toward the hydroxyl substrate glycerol

**Figure 1:**
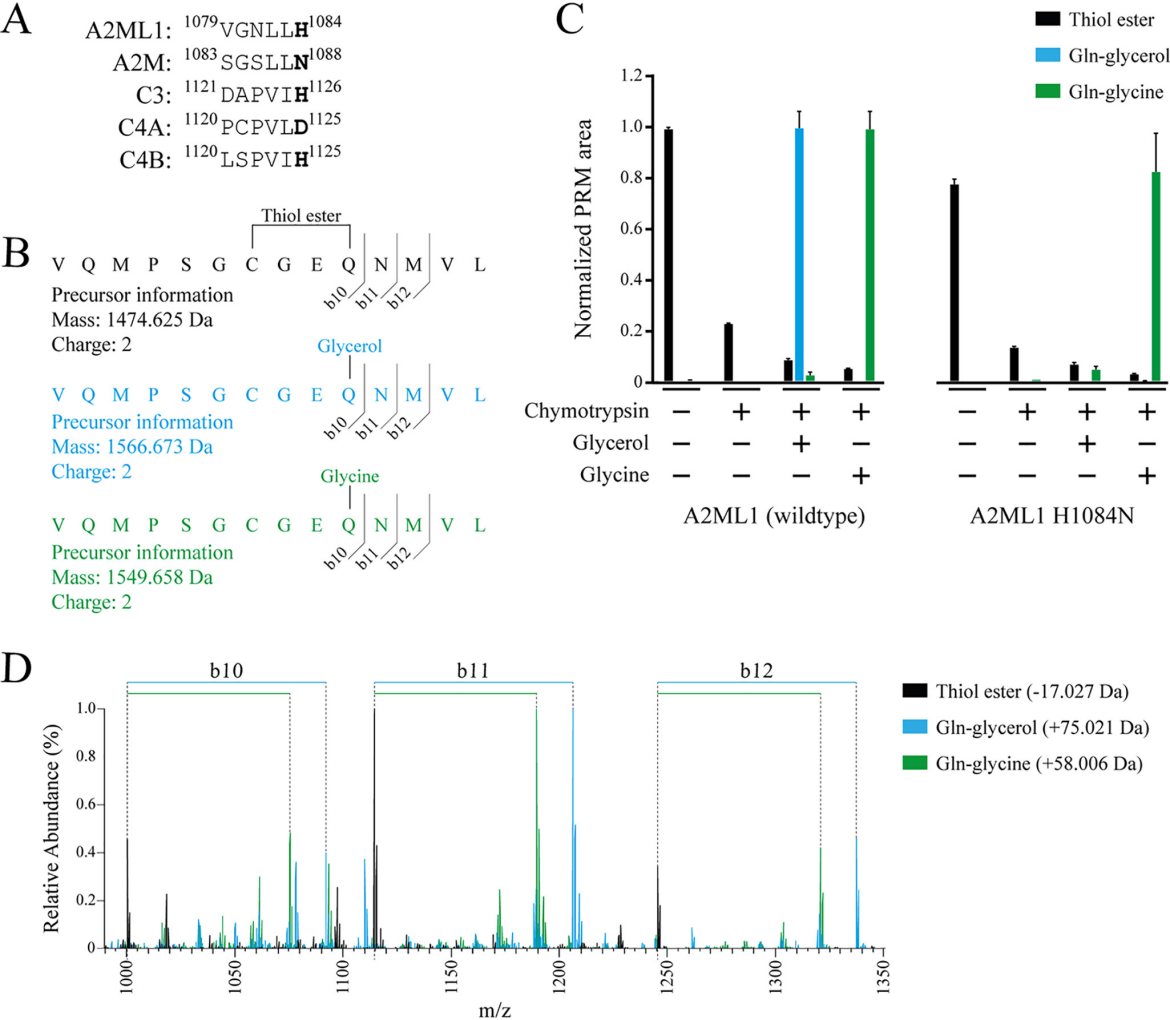
**Reactivity of A2ML1's thiol ester toward amine and hydroxyl substrates.***A*, sequence alignment of A2ML1 and four other αM proteins showing the hydroxyl reactivity–conveying histidine possessed by A2ML1, C3, and C4B. *B*, WT and H1084N A2ML1 were cleaved by chymotrypsin in HBS with 200 mm of glycerol or glycine. The samples were acidified with formic acid to pH 3, digested using pepsin, and analyzed by LC-MS/MS. The major peptide covering the thiol ester site, with either an intact thiol ester or after conjugation to glycerol or glycine, was quantified by PRM. The fragmentation sites that produce the b10, b11, and b12 product ions used for quantification are indicated. *C*, PRM was used to quantify the peptide covering the thiol ester site with the three modifications. The sums of the b10, b11, and b12 product ion areas of each modified peptide were normalized to the sample in which that peptide was most abundant. Both A2ML1s were able to conjugate glycine, but formation of the glycerol reaction product was only detected using WT A2ML1. Quantification is based on three separately prepared samples per condition, and the *error bars* indicate the S.D. *D*, overlaid MS2 spectra from the three peptides. The b10, b11, and b12 product ions that were used for PRM quantification from each spectrum are indicated. As these product ions include the glutamine that participates in the thiol ester (Gln-973), their mass is increased by the mass of a glycerol or glycine after conjugation. The full annotated MS2 spectra for each peptide are shown in Fig. S1.

To establish whether His-1084 conveys hydroxyl reactivity of A2ML1's thiol ester, as the equivalent histidine residues do in C3 and C4B (Fig. 1*A*), the formation of glycine and glycerol conjugation products by recombinantly expressed WT A2ML1 and the A2ML1 H1084N mutant were measured using LC-MS/MS. The two A2ML1s were proteolytically activated using chymotrypsin in the presence of 200 mm glycine or glycerol, or buffer only; the A2ML1s without proteolytic activation, and therefore with intact thiol esters, were also included. The samples were then acidified to pH 3 and digested using pepsin; this low-pH digest prevents nucleophilic attack of the thiol ester and allows it to persist until LC-MS/MS, which also takes place at low pH. Initial LC-MS/MS studies were used to identify the major peptide covering the thiol ester site (Table S1 and Fig. S1). A parallel reaction monitoring (PRM) experiment was then performed to quantify this peptide modified with either an intact thiol ester or a Gln-glycine or Gln-glycerol group formed by reaction of the thiol ester with either substrate (Fig. 1, *B*–*D*). Although both WT and H1084N A2ML1s reacted with the amine substrate glycine, the reaction product with the hydroxyl substrate glycerol was only detected for WT A2ML1 (Fig. 1, *B*–*D*), indicating that His-1084 conveys hydroxyl reactivity in A2ML1. We note that the abundance of a given peptide can be compared across samples using MS, but that the abundance of different peptides within the same sample are not comparable because of their potentially different ionizabilities. Therefore, the relative reactivity of WT A2ML1 toward the two substrates was not defined.

### WT A2ML1 can conjugate to proteases through its hydroxyl side chains

**Figure 2:**
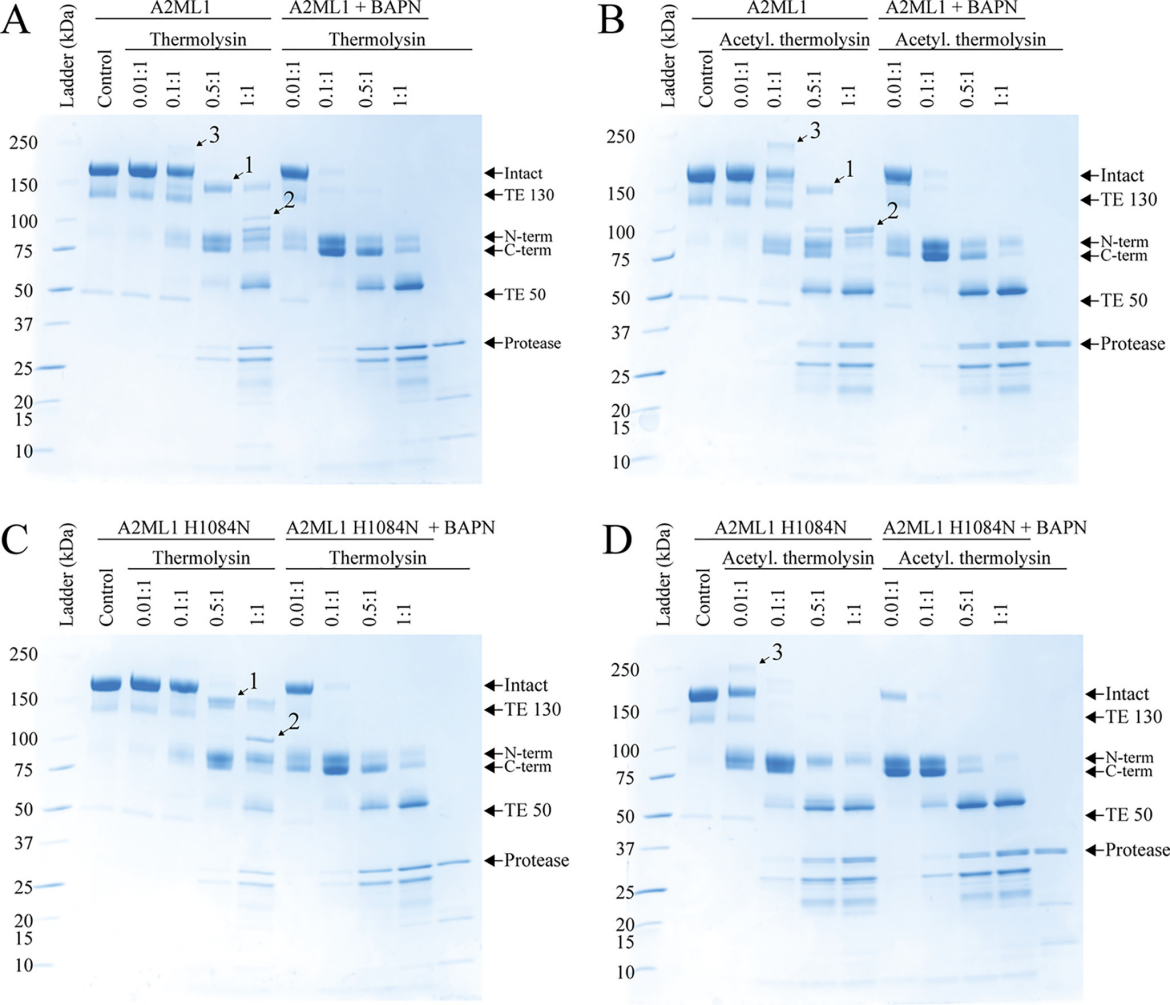
**Reducing SDS-PAGE of A2ML1 digested with thermolysin.***A*–*D*, WT (*A* and *B*) and H1084N (*C* and *D*) A2ML1 were digested with unmodified (*A* and *C*) or acetylated (*B* and *D*) thermolysin at various molar ratios of thermolysin:A2ML1, as indicated, for 15 min at 37°C, after which thermolysin was inhibited with 10 mm EDTA. Digestions were also performed with 50 mm BAPN present, which competes with proteases for thiol ester–mediated conjugation. An intact A2ML1 protein migrates as an ∼180-kDa band. When an intact thiol ester is present, some A2ML1 is fragmented when heated under denaturing conditions to ∼130-kDa and ∼50-kDa bands. Bait region cleavage gives an N-terminal cleavage product of ∼95 kDa and a C-terminal cleavage product of ∼85 kDa. The C-terminal cleavage product includes the thiol ester site and participates in protease conjugation products, which for thermolysin are ∼150 kDa and are indicated as *band 1*. This conjugation product can be further cleaved by thermolysin to yield *band 2*. In-gel digestion and MS were used to verify that *bands 1* and *2* contain both A2ML1 and thermolysin. A third band, *band 3*, contains both the N-terminal and C-terminal fragments of A2ML1, but not thermolysin, and is because of intra-A2ML1 thiol ester conjugation, as shown in Fig. S4. Both A2ML1s are initially cleaved by thermolysin in their bait regions, with secondary cleavage elsewhere occurring at higher thermolysin ratios. Both A2ML1s form conjugation products to unmodified thermolysin, which has both hydroxyl and amine groups, but only WT A2ML1 conjugates to acetylated thermolysin, which only has hydroxyl groups. Digestion proceeds more readily in circumstances when protease conjugation is not possible, *e.g.* because of BAPN or when A2ML1 H1084N is cleaved by acetylated thermolysin.

We proceeded to investigate the ability of WT and H1084N A2ML1s to form thiol ester–mediated conjugation products with proteases with and without accessible primary amines (*i.e.* their N termini and lysine side chains). First, we used reducing SDS-PAGE to identify bands corresponding to conjugation products between either A2ML1 and unmodified thermolysin, which has 11 lysine residues, or thermolysin which had been acetylated using amine-reactive chemistry (Fig. 2). The 70-Da primary amine–containing compound BAPN was used to compete with proteases for thiol ester–mediated conjugation, to better define the conjugation product bands. Both A2ML1s formed an ∼150-kDa migrating conjugation product between unmodified thermolysin (34 kDa) and the C-terminal fragment of bait region–cleaved A2ML1 (82 kDa without glycans) (*band 1*, Fig. 2, *A* and *C*). Further proteolysis of this primary conjugation product gives an ∼100-kDa migrating conjugation product (*band 2*, Fig. 2, *A* and *C*). Using acetylated thermolysin, these conjugation products were also formed by WT A2ML1 (Fig. 2*B*), but not A2ML1 H1084N (Fig. 2*D*). LC-MS/MS analysis of in-gel digested bands was used to confirm that these bands contained both A2ML1 and thermolysin. The dependence of A2ML1 H1084N but not WT A2ML1 on primary amines to conjugate proteases was also demonstrated using human neutrophil elastase (HNE), which has no lysine residues and whose N terminus is structurally buried and therefore inaccessible (25, 26), and cathepsin G (CatG), which has four lysine residues (Fig. S2). These results indicated that A2ML1 can conjugate to proteases lacking accessible primary amine groups in a manner that depends on its hydroxyl reactivity, which is conveyed by His-1084.

**Figure 3:**
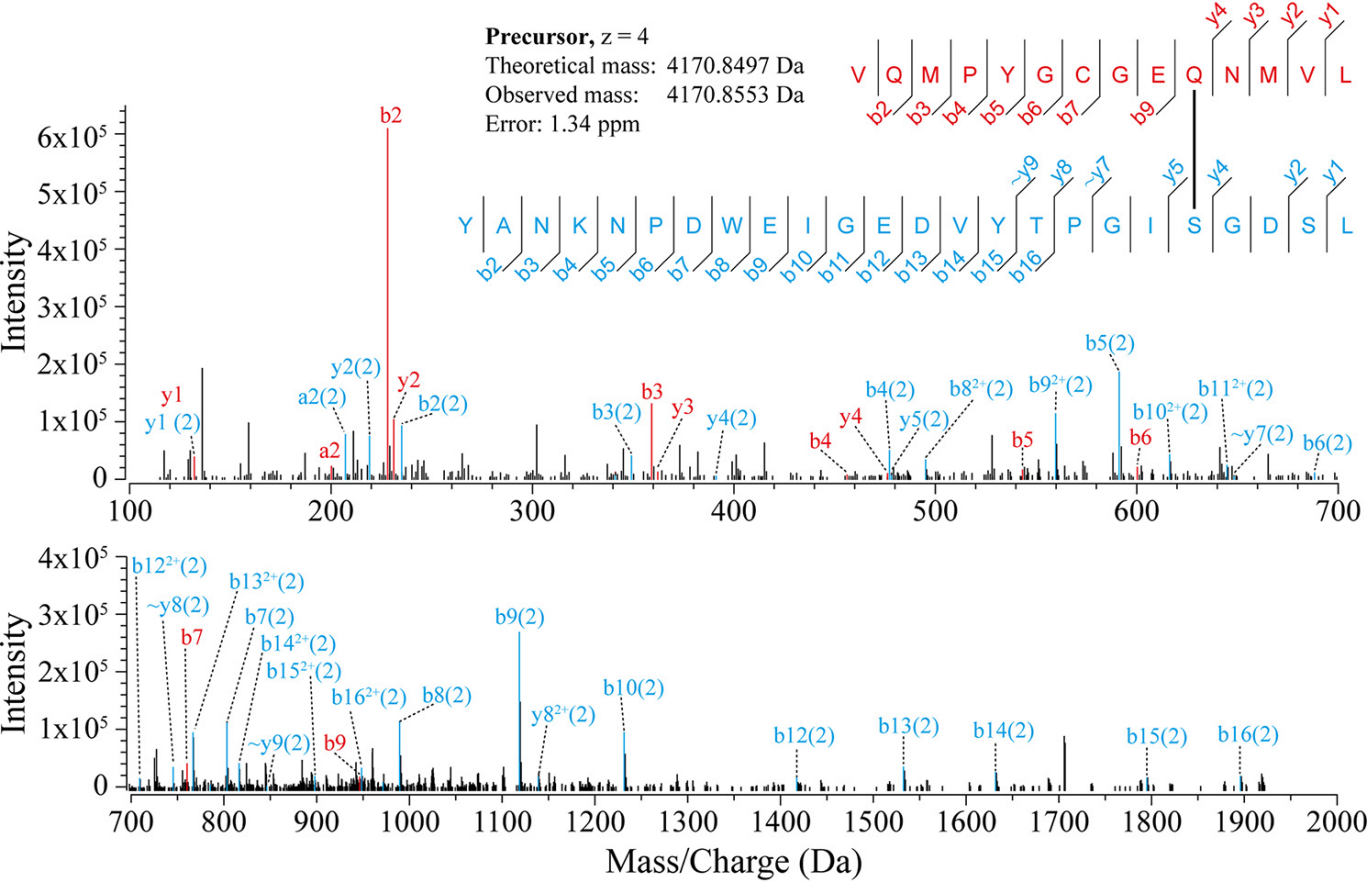
**An MS2 spectrum identifying conjugation of A2ML1's thiol ester to a thermolysin serine side chain.** The SDS-PAGE band of the conjugation product between unmodified thermolysin and the C-terminal fragment of bait region–cleaved WT A2ML1, *i.e. band 1* in Fig. 2, was digested with pepsin, extracted from the gel band, and analyzed by LC-MS/MS. Crosslinks between the thiol ester–covering peptide (VQMPYGCGEQNMVL) and thermolysin peptides were identified. In the above MS2 spectrum, HCD-fragmented products from the thiol ester peptide (in *red*) and a thermolysin peptide (in *blue*) cover both ends of each peptide and an extensive b product ion series (b4–b16) shows the lysine residue on the thermolysin peptide, K4, as unmodified. Fig. S3 conclusively demonstrates that K4 is not the site of conjugation, as the same experiment using acetylated thermolysin identified a crosslinked peptide identical to this one, except for acetylation of K4. Note that the ∼y7, ∼y8, and ∼y9 fragments show the putative site of conjugation S20 as unmodified. The other hydroxyl residues Y1, Y15, T16, and S23 are seen unmodified in fragments b2–b16, b15, b15/b16/y9, and y1/y2, respectively, and there are therefore no conjugation site candidate positions that are not seen as unmodified residues. However, the y8^2+^ residue shows S20 crosslinked to the thiol ester peptide and thus supports S20 as the conjugation site. The spectra of additional crosslinked peptides are given in Fig. S3.

By digesting the gel band corresponding to the ∼150-kDa conjugation product between WT A2ML1 and thermolysin with pepsin and analyzing the resulting peptides with LC-MS/MS, crosslinks between the thiol ester peptide of A2ML1 and various thermolysin peptides could be identified (Fig. 3 and Fig. S4). In several cases, both lysine and hydroxyl (serine, threonine, and tyrosine) side chains were present on the thermolysin peptide and fragment coverage was insufficient to determine the precise site of conjugation (Fig. S4). However, one crosslinked peptide was detected for both unmodified and acetylated thermolysin, ruling out its lysine residue and specifying a serine residue as the site of conjugation (Fig. 3). This directly shows that WT A2ML1 can conjugate proteases through hydroxyl side chains.

In addition to its conjugation products with proteases, both A2ML1s also formed an ∼250-kDa conjugation product following proteolytic activation that did not contain any protease (*band 3*, Fig. 2); this band is especially prominent when A2ML1 is cleaved by the *Streptococcus aureus* glutamyl endoprotease, GluC, which it is not able to conjugate (Fig. S2). This product depends on thiol ester–mediated conjugation, as it disappears upon the addition of BAPN (Fig. S3). Nonreducing SDS-PAGE showed that the conjugation happens within a single A2ML1 (Fig. S3). Both the N-terminal and C-terminal bait region–cleaved fragments of A2ML1 were detected in LC-MS/MS of the in-gel–digested band and Edman sequencing of the band. We conclude that this band constitutes an intra-A2ML1 conjugation product between the thiol ester and one or more undetermined sites in the N-terminal bait region–cleaved A2ML1 fragment, and likely occurs sporadically when A2ML1 fails to trap a protease by which it has been cleaved.

**Figure 4:**
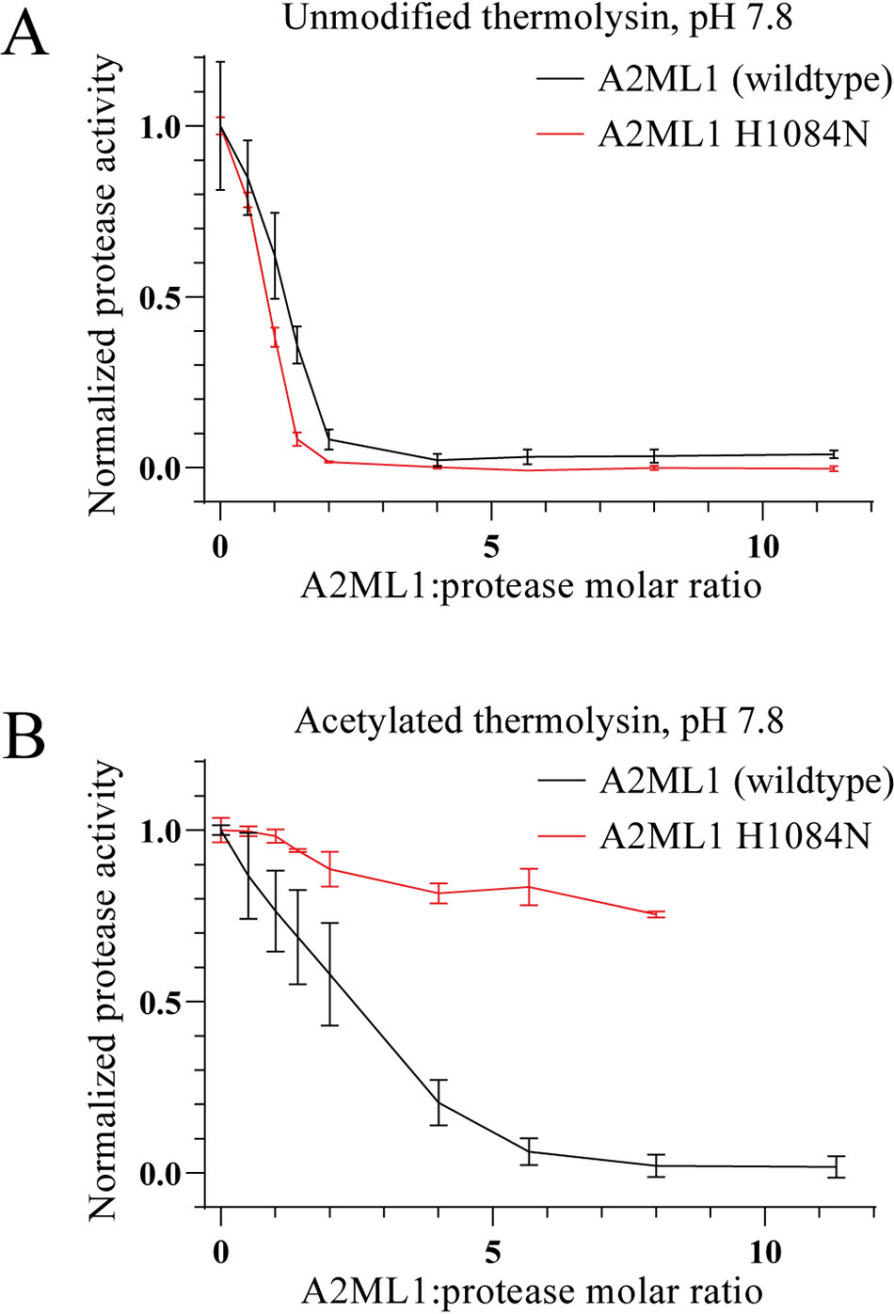
**Inhibition of thermolysin by A2ML1.***A* and *B*, unmodified (*A*) and acetylated (*B*) thermolysin were incubated with the indicated ratios of A2ML1 or A2ML1 H1084N for 15 min at 37°C in 150 mm Tris-HCl, 15 mm CaCl_2_, pH 7.8. The samples were then incubated for an additional 60 min at 37°C with resorufin-labeled casein, after which TCA was added to terminate the reactions and precipitate intact substrate. Precipitated substrate was removed by filtration. The filtrate was neutralized and the residual proteolytic activity was determined by measuring the absorbance at 574 nm. *n* = 3 and the *error bars* show the S.D. Both A2ML1s inhibited unmodified thermolysin, requiring roughly a 2-fold excess of A2ML1 for full inhibition. WT A2ML1 inhibited acetylated thermolysin, albeit less efficiently, requiring a roughly 6-fold excess of A2ML1 for full inhibition, whereas A2ML1 H1084N demonstrated little to no inhibition.

Following the SDS-PAGE analysis of thermolysin-cleaved A2ML1, we assessed the ability of WT and H1084N A2ML1 to inhibit the cleavage of casein by unmodified and acetylated thermolysin. Both A2ML1s were able to inhibit unmodified thermolysin, requiring an approximately 2-fold molar surplus of A2ML1 for full inhibition (Fig. 4*A*). WT A2ML1 was able to inhibit acetylated thermolysin, although this inhibition was less efficient than for unmodified thermolysin and required an approximately 6-fold molar surplus of A2ML1 for full inhibition (Fig. 4*B*). In contrast, little to no inhibition of acetylated thermolysin by A2ML1 H1084N was seen, even at an 8-fold molar surplus of A2ML1 (Fig. 4*B*). These results show that WT A2ML1's conjugation of proteases through hydroxyl side chains is associated with protease inhibition.

### A2ML1's hydroxyl reactivity does not increase protease conjugation or inhibition at pH 5

**Figure 5:**
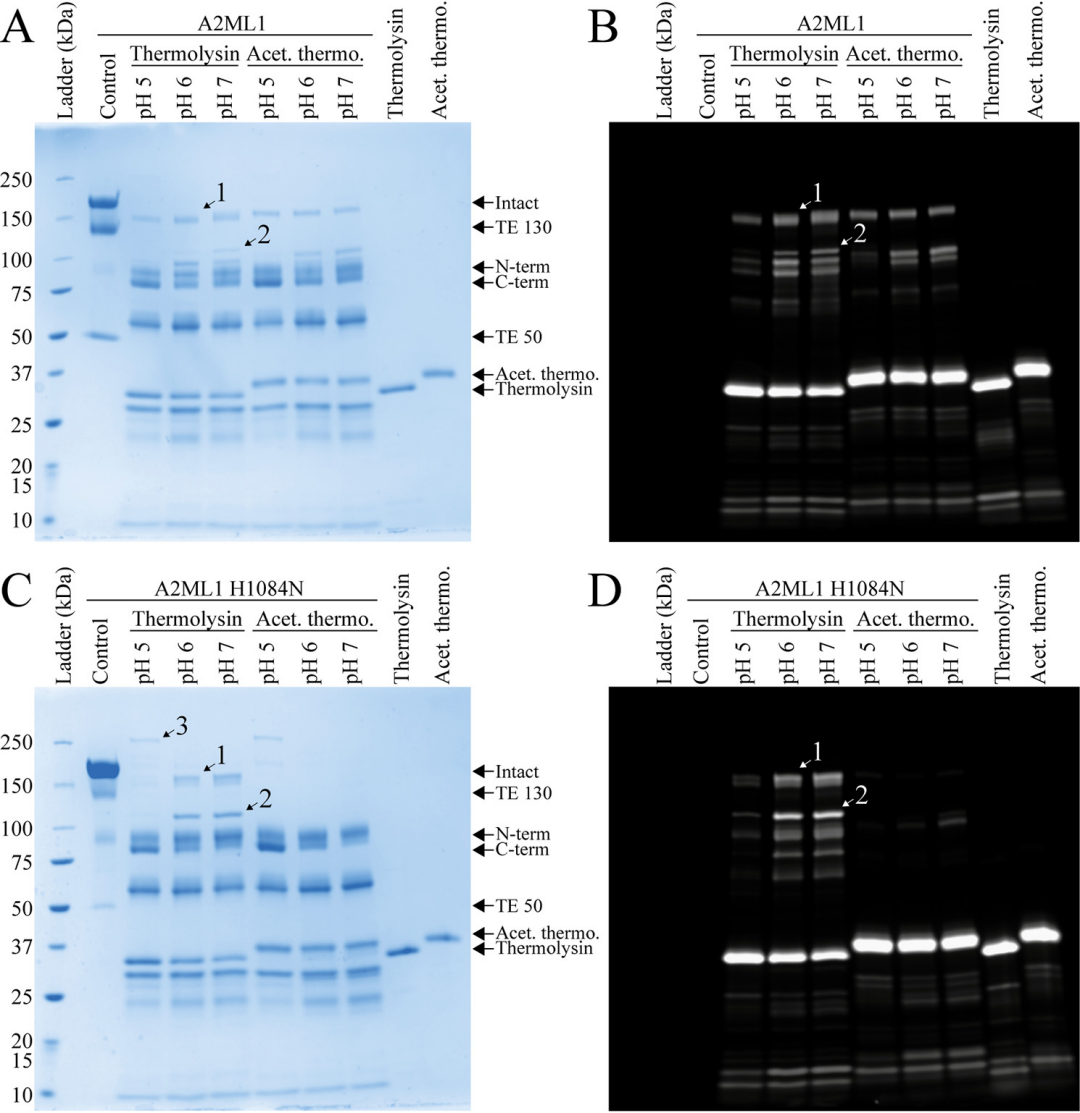
**SDS-PAGE analysis of A2ML1's conjugation to Cy5-labeled thermolysin at pH 5–7.***A*–*D*, WT (*A* and *B*) and H1084N (*C* and *D*) A2ML1 were digested using a 1:1 molar ratio of thermolysin that was either only Cy5-labeled or was additionally acetylated after Cy5 labeling. Digestion was performed at pH 5, 6, or 7. Fluorescence images from 20 s of exposure visualizing the bands containing thermolysin (*B* and *D*) and images of the gels after Coomassie Blue staining (*A* and *C*) are shown. The major conjugation product between thermolysin and A2ML1 is labeled *band 1*, further processing of this product gives *band 2*, and intra-A2ML1 conjugation gives *band 3*, using the same numbering as in Fig. 2. Although both A2ML1s conjugate thermolysin similarly at pH 6 and 7, the ∼150 kDa major conjugation product (*band 1*) is moderately more intense at pH 5 for WT A2ML1 than for A2ML1 H1084N. Furthermore, acetylated thermolysin, which is only conjugated by WT A2ML1, yields a similarly intense conjugation product at pH 5 to those at pH 6 and 7, although it should be noted that secondary cleavage of the major conjugation product (*e.g.* yielding *band 2*) occurs to a lesser degree at pH 5 because of the lower activity of thermolysin at this pH, and the amount of ∼150 kDa conjugation is therefore not solely indicative of the degree to which conjugation takes place.

We hypothesized that A2ML1's reactivity toward hydroxyl groups might be advantageous in the epidermal environment, as the lower pH of the epidermis might affect the nucleophilicities of hydroxyl and amine groups differently. To test this, we used reducing SDS-PAGE to assess the formation of conjugation products between both A2ML1s and both thermolysins at pH 5, 6, and 7 (Fig. 5, *A*–*D*). For this experiment, both thermolysins were Cy5-labeled, which allows conjugation products to be identified by their fluorescence (Fig. 5, *B* and *D*). Both A2ML1s showed a similarly decreased conjugation of unmodified thermolysin as the pH was lowered, indicating that hydroxyl reactivity did not increase conjugation at lower pH (Fig. 5). Consistent with previous results, only WT A2ML1 was able to conjugate acetylated thermolysin (Fig. 5). The inhibition of unmodified thermolysin's cleavage of casein by either A2ML1 was less efficient at pH 5 than at pH 7.8, as shown in Fig. 4, requiring a roughly 4-fold surplus of A2ML1 for full inhibition and with no significant difference between the A2ML1s (Fig. S5). We conclude that A2ML1's hydroxyl reactivity does not improve its conjugation efficiency or protease inhibition at lowered pH values.

### The N1088H mutation does not significantly increase A2M's conjugation of proteases through hydroxyl side chains

**Figure 6:**
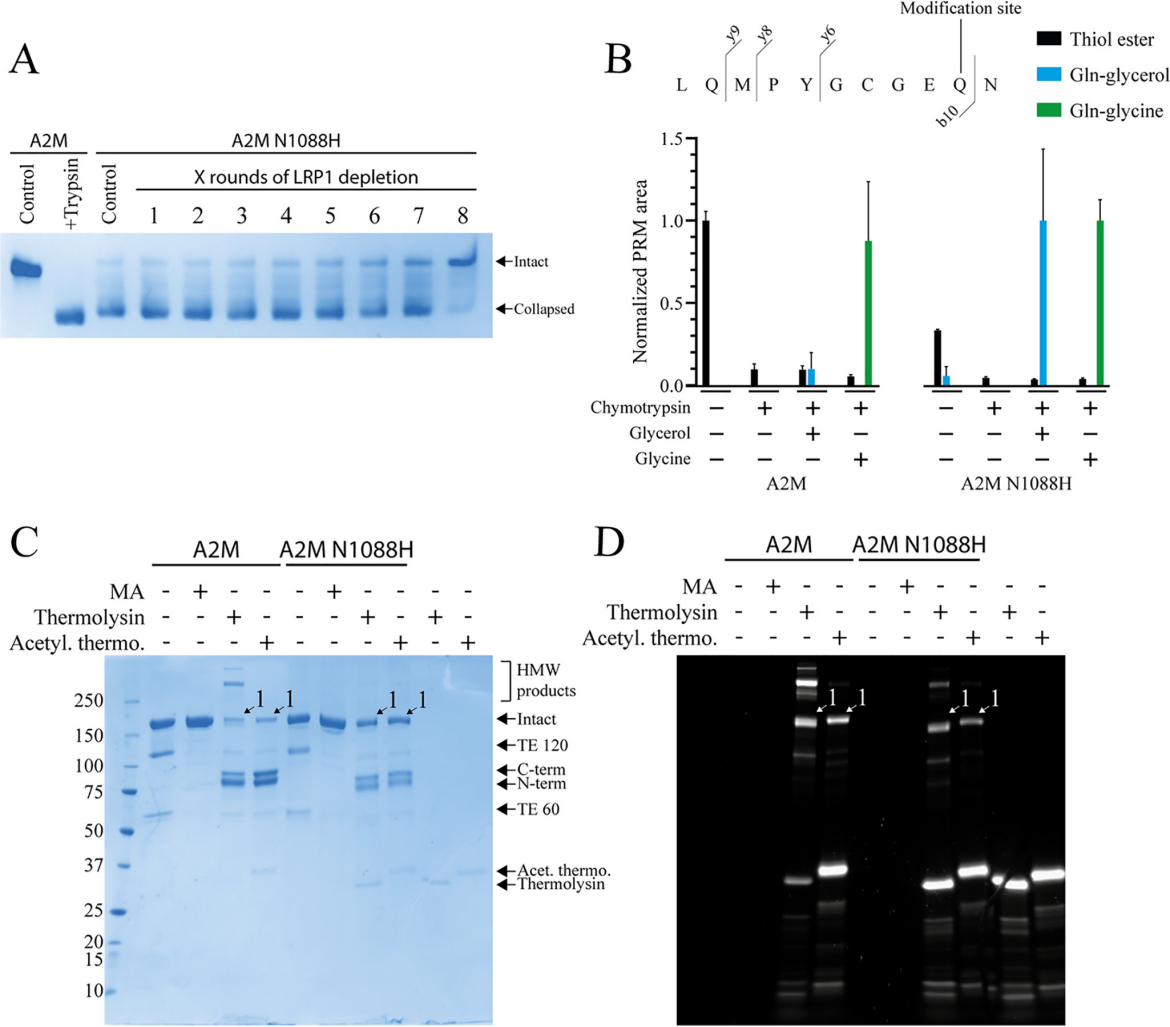
**Thiol ester reactivity of A2M N1088H.** The mutant A2M N1088H, in which the histidine conveying hydroxyl reactivity A2ML1 is introduced, was recombinantly expressed and characterized. *A*, pore-limited native PAGE of A2M N1088H before and after up to eight rounds of depletion using LRP1-loaded resin. Plasma-purified A2M in its intact native conformation and trypsin-cleaved collapsed conformation is included for comparison. A2M N1088H was expressed mostly in a collapsed conformation. However, the collapsed A2M N1088H could be depleted by using its binding to LRP1, allowing native A2M N1088H to be enriched. *B*, the reactivity of the plasma-purified and N1088H A2M thiol esters toward glycerol and glycine, as determined using MS PRM. The same approach used for A2ML1, as described in Fig. 1, was used for A2M. PRM quantification was based on the b10, y6, y8, and y9 fragments that cover Gln-975, which participates in the thiol ester and becomes conjugated to glycerol or glycine. Both A2Ms reacted similarly with the amine substrate glycine, but A2M N1088H showed an increased reactivity toward the hydroxyl substrate glycerol. Quantification is based on three separately prepared samples per condition, and the *error bars* indicate the S.D. *C* and *D*, reducing SDS-PAGE of plasma-purified and N1088H A2M treated with methylamine or cleaved with thermolysin that was either only Cy5-labeled or was additionally acetylated after Cy5 labeling. The Coomassie Blue–stained gel (*C*) and Cy5 fluorescence image (*D*) are shown. The conjugation product between the C-terminal bait region cleaved fragment of A2M and thermolysin is indicated as *band 1*, although this band co-migrates with that of the intact A2M subunit. Both A2Ms conjugated thermolysin and, to a lesser extent, acetylated thermolysin. A2M N1088H did not demonstrate an increased relative conjugation to acetylated thermolysin as compared with plasma-purified A2M.

We investigated the effect of the N1088H mutation on human A2M, which introduces a histidine residue at the position equivalent to the hydroxyl reactivity–conveying histidine of A2ML1, C3, and C4B. A2M N1088H was mostly expressed in its collapsed conformation by suspension-adapted HEK293 cells (Fig. 6*A*), in contrast to WT recombinant A2M which is almost entirely expressed in its native conformation.^3^ The glutamine residue Gln-975, which participates in the thiol ester of A2M, was seen as unmodified in LC-MS/MS of A2M N1088H. Deamidated Gln-975, which would be formed by thiol ester hydrolysis, was not detected. This indicates that the N1088H mutation compromised A2M's ability to form the thiol ester. Taking advantage of the affinity of collapsed A2M, but not native A2M, for the scavenger receptor LRP1, we depleted the collapsed A2M N1088H using a resin loaded with cluster 1B of LRP1, which is the fragment that binds to A2M (28), to enrich native A2M N1088H. After eight rounds of LRP1 depletion, the remaining A2M N1088H was mostly native (Fig. 6*A*). We proceeded to identify the major peptide covering the thiol ester in pepsin-digested A2M (Table S1 and Fig. S6), allowing us to design a PRM experiment to compare the hydroxyl reactivity of WT A2M purified from plasma and A2M N1088H. The formation of reaction products between the thiol ester and glycerol was detected for A2M N1088H but not WT A2M, whereas both A2Ms were able to conjugate glycine (Fig. 6*B*). Both A2Ms conjugated unmodified thermolysin and, to a lesser extent, acetylated thermolysin, as determined by reducing SDS-PAGE using Cy5-labeled thermolysin (Fig. 6*C*). However, the relative conjugation of acetylated and unmodified thermolysin was very similar for both A2Ms, indicating that the N1088H mutation conveys little to no ability to conjugate proteases through their hydroxyl residues and that the increased hydroxyl reactivity of A2M N1088H seen in the PRM experiment is likely to be only a minor increase relative to WT A2M.

## Discussion

Protease inhibition by A2ML1 has been demonstrated *in vitro* in a single previous publication (1). As such, the physiological relevance of A2ML1 as a protease inhibitor is not well-established, and the function of A2ML1's hydroxyl reactivity–conveying histidine, which is normally associated with αM complement factors, raises further questions. In this study, we show that A2ML1's hydroxyl reactivity is in fact advantageous for the conjugation and inhibition of proteases that lack accessible primary amine groups. Furthermore, conjugation to both hydroxyl and amine groups is possible when A2ML1 is bait region–cleaved by proteases with a normal surface distribution of lysine residues. We propose that A2ML1's hydroxyl reactivity facilitates the physiological trapping of one or more currently unidentified proteases that lack lysine side chains that are accessible for thiol ester–mediated conjugation. Hydroxyl reactivity allows conjugation to instead take place through hydroxyl groups on side chains or glycans. Several candidates can be proposed on the basis of their primary sequence alone; for example, HNE has no lysine residues and activated kallikrein 12 has only a single lysine residue. We show in this study that HNE can be conjugated by A2ML1 in a manner that depends on His-1084, and A2ML1 may encounter active HNE during inflammation *in vivo*. However, it remains possible that A2ML1's hydroxyl reactivity is vestigial or a neutral trait, or it may have a function outside of the context of protease inhibition, for example in complement-like membrane deposition of A2ML1. Further investigation of the physiological role of A2ML1 is required to clarify this issue.

We investigated whether hydroxyl reactivity could be conveyed to A2M by the N1088H mutation similarly to how it could be removed from A2ML1 through the H1084N mutation. However, the N1088H mutation severely compromised the ability of A2M to form its thiol ester, resulting in the majority of A2M N1088H being found in a collapsed conformation. This likely results from perturbation of the interface between A2M's thiol ester and receptor-binding domains, which maintain a hydrophobic environment in which the thiol ester is spontaneously formed (11, 29, 30). Although the remaining native A2M N1088H had an increased reactivity toward glycerol, both A2Ms were able to conjugate acetylated thermolysin, and A2M N1088H did not show a significantly increased conjugation through non-amine groups. These results indicate that the reactivity of the thiol ester toward hydroxyl groups is affected by additional factors beyond the presence of an acyl-imidazole–forming histidine, which is required for A2ML1 but not A2M to conjugate acetylated thermolysin. Indeed, WT A2M can conjugate tyrosine side chains on thermolysin through ester bonds (14). It is possible that the ability of A2M to trap proteases noncovalently in its hollow interior increases the window of opportunity for thiol ester–mediated conjugation.

Regardless, as A2M does not require covalent conjugation to trap proteases (20), an acyl-imidazole–forming histidine is likely of little benefit even if it could be incorporated without functional disruption of A2M. In contrast, A2ML1 appears to require covalent conjugation to proteases to inhibit them, as is the case with other monomeric αM protease inhibitors such as α_1_-I_3_ and ECAM (15, 16), meaning that a broadened thiol ester reactivity corresponds to an expanded repertoire of proteases that can be conjugated and inhibited. This study demonstrates a role for histidine-enhanced hydroxyl reactivity in protease inhibition by A2ML1 and potentially other αM protease inhibitors.

## Materials and methods

### Expression and purification of recombinant proteins

A plasmid for recombinant A2ML1 expression was made by synthesizing the consensus coding sequence A8K2U0-1 (http://www.rcsb.org/pdb/protein/A8K2U0), flanked by the 5′ and 3′ untranslated regions of A2M mRNA M11313 (32), and cloning it into the pCDNA3.1(+) plasmid using NheI and XbaI restriction sites. Likewise, the plasmid encoding recombinant A2M was prepared by synthesizing a WT A2M gene based on the mRNA sequence M11311 (32) and inserting it into the pCDNA3.1(+) plasmid using the NheI/XbaI restriction sites. The H1084N and N1088H mutations of A2ML1 and A2M, respectively, were implemented by site-directed mutagenesis. A gene for LRP1 cluster 1B (residues 20 to 974 of human LRP1, pre-pro numbering) was synthesized in tandem with an N-terminal StrepII double tag and a C-terminal Fc region from human IgG1 and inserted into pcDNA3.1(+). All gene synthesis, cloning, and mutagenesis was performed by GenScript.

A2ML1 was expressed in suspension-adapted HEK293 Freestyle cells using a standard protocol. Briefly, plasmid DNA and 25 kDa linear polyethyleneimine (Polysciences Inc.) were mixed at a 1:4 w/w ratio in antibiotic-free Freestyle medium (Thermo Fisher Scientific) and incubated for 10 min. This was then dripped into a cell culture at a concentration of 1 million cells per ml to a final DNA concentration of 1 μg per ml. The cells were cultured for 3 days, at which point the supernatant was harvested by removing the cells through centrifugation.

A2ML1 was purified from the conditioned supernatant in a three-step process using sequential Zn^2+^ immobilized metal ion affinity chromatography on a HiTrap Chelating HP column (GE Healthcare), anion exchange chromatography on a HiTrap Q (GE Healthcare), and size exclusion chromatography on a Superdex 200 Increase (GE Healthcare). All steps were performed on an ÄKTA Purifier system (GE Healthcare).

Plasma-purified and recombinant A2M were prepared as described previously.^3^ Cluster 1B of LRP1, which binds to A2M (28), was expressed in fusion with a twin StrepII tag and the Fc region of human IgG1, and purified using affinity chromatography with a Strep-Tactin column (IBA GmbH).

### Acetylation and Cy5 labeling of thermolysin

Thermolysin from *Geobacillus stearothermophilus* (Sigma-Aldrich) was acetylated using a 10:1 w/w surplus of sulfo-NHS-acetate (Thermo Fisher Scientific) for half an hour at room temperature in 100 mm triethylammonium bicarbonate (TEAB), pH 8.3. The reaction was quenched with 50 mm Tris-HCl, pH 8, and the acetylated thermolysin was desalted into HBS with 5 mm CaCl_2_ using a PD-10 column (GE Healthcare).

Cy5-labeled thermolysin was prepared by labeling thermolysin with a 1:1 molar ratio of Cy5 mono NHS ester (GE Healthcare) in 100 mm TEAB, pH 8.3, for half an hour at room temperature. The reaction was quenched with Tris-HCl and the Cy5-labeled thermolysin was desalted as for acetylated thermolysin. Absorbance at 280 nm and 550 nm was used to calculate the labeling ratio, which was an ∼0.6:1 molar ratio of Cy5 to thermolysin. A portion of the Cy5-labeled thermolysin was then acetylated as described for unlabeled thermolysin.

### MS-based quantification of reaction products between thiol esters and glycine or glycerol

The reaction products between glycine or glycerol and the exposed thiol esters of A2ML1 (WT and H1084N mutant) and A2M (purified from plasma or N1088H) were analyzed by pepsin digestion and LC-MS/MS. 1.5 μg of protein were mixed with HEPES-buffered saline only (HBS, 20 mm HEPES, 150 mm NaCl, pH 7.4) or with pH 7.4 glycine or glycerol added to a final concentration of 200 mm. Thiol esters were then proteolytically activated by adding chymotrypsin to a 1.2:1 protease:A2ML1 molar ratio or a 2.4:1 protease:A2M molar ratio. Samples were incubated for 30 min at 37°C, after which the digestion and conjugation reactions were quenched by adding formic acid (FA) to acidify the samples to pH 3. The samples were digested by pepsin at a 1:20 w/w ratio overnight at 37°C. Peptides from the digested samples were then purified using pipette tips packed with POROS 50 R2 C18 resin (PerSeptive Biosystems). Samples were prepared in triplicate.

In each run, 250 nanograms of peptide were analyzed by LC-MS/MS. Online reverse-phase HPLC (RP-HPLC) was done on an EASY-nLC 1200 (Thermo Fisher Scientific) with a 5–45% gradient of acetonitrile (ACN) over 50 min. MS was performed on an Orbitrap Eclipse Tribrid (Thermo Fisher Scientific) running in data-dependent acquisition mode; peptides were isolated at their elution apex and fragmented by high-energy collision dissociation (HCD) with stepped energies (27, 30, and 32%). The resulting MS data were analyzed using the Byonic search engine (version 3.7.13, Protein Metrics), using a sequence database containing only the protein of interest (A2ML1 or A2M). The searches used variable modification of glutamine with either an intact thiol ester (−NH_3_), an ester bond to glycerol (+C_3_H_5_O_3_N_−1_), or an amide bond to glycine (+C_2_H_2_O_2_). Protease cleavage was set to nonspecific. Mass tolerance was set to 10 ppm for both precursor peptides and MS^2^ fragments. Peptide spectral matches to the A2ML1 thiol ester peptide VQMPSGCGEQNMVL or the A2M thiol ester peptide LQMPYGCGEQN with these three modifications were manually assessed (without FDR or score requirements) and were used to design a PRM study to quantify the thiol ester peptide and its reaction products with glycine and glycerol in the samples. Peptide information is given in Table S1 and peptide spectra for A2ML1 and A2M are shown in Figs. S1 and S6, respectively. The targeted PRM study was performed using the same equipment, LC gradient, and fragmentation method as described for LC-MS/MS with data-dependent acquisition. PRM data were quantified using Skyline version 20.1.0.155 (33); using the b10, b11, and b12 product ions from the VQMPSGCGEQNMVL peptide in the A2ML1 study; and using the y6, y8, y9, and b10 products ions from the LQMPYGCGEQN peptide in the A2M study. The Skyline files have been made available; see “Data availability” for details.

### SDS-PAGE

Denaturing SDS-PAGE was performed using the discontinuous ammediol/glycine buffer system on homemade 5–15% acrylamide gradient gels (34). When noted, samples were reduced with 25 mm DTT for 5 mins at 95°C. Native pore limited PAGE was performed as described previously (27), using homemade gels in TBE buffer (89 mm Tris, 89 mm boric acid, 2 mm EDTA) with an acrylamide gradient of 5–10%.

### Proteolytic digests of A2ML1

Digestions with HNE, CatG, thermolysin, and acetylated thermolysin were done with up to a 1:1 molar ratio of protease to WT or H1084N A2ML1. All of the reactions were carried out in HBS with and without 50 mm BAPN. 5 mm CaCl*_2_* was added to the reactions with thermolysin and acetylated thermolysin. The reaction mixes were incubated for 15 min at 37°C, after which digestion was stopped by inhibiting HNE and CatG with 2 mm PMSF for 15 min or inhibiting thermolysin and acetylated thermolysin with 10 mm EDTA for 15 min.

Similar reactions were carried out at pH 5, 6, and 7 using Cy5-labeled thermolysin and Cy5-labeled acetylated thermolysin at a 1:2 molar ratio of protease to A2ML1. These reactions were performed in 50 mm sodium acetate, pH 5, 6, or 7, 137 mm NaCl, and 5 mm CaCl_2_.

### Identification of proteins in SDS-PAGE bands using Edman degradation and LC-MS/MS

To identify the proteins present in suspected protease conjugation product bands using Edman degradation, samples of WT A2ML1 incubated with a 1:1 molar ratio of HNE, cathepsin G, and unmodified thermolysin were separated by reducing SDS-PAGE and transferred to a PVDF membrane. The conjugation product bands were cut out and applied to TFA-treated glass fiber membranes. Automated Edman degradation was performed on a PPSQ-31B protein sequencer (Shimadzu Biotech) with in-line phenylthiohydantion analysis on an LC-20AT HPLC system. Data were obtained using Shimadzu PPSQ-31B software and the sequences were determined manually from the UV 269 nm chromatograms.

To identify the proteins present in suspected protease conjugation product bands using LC-MS/MS, gel bands prepared as described for Edman degradation were excised, shrunk with ACN, and swelled with 50 mm ammonium bicarbonate, pH 8, 25 ng/ml MS-grade trypsin (Thermo Fisher Scientific). Digestion was performed overnight at 37°C. The digested peptides were purified using homemade C18 stage tips and analyzed by LC-MS/MS on a Q Exactive Orbitrap (Thermo Fisher Scientific). The data were searched using the Mascot search engine (version 2.5) with fully tryptic protease cleavage, using cysteine propionamidylation as a fixed modification, methionine oxidation and glutamine/asparagine deamidation as variable modifications, and with mass tolerances of 10 ppm for precursors and 0.2 Da for MS^2^ fragment ions. The sequence database used was SwissProt (updated January 2020), restricted to human and bacterial sequences. Peak lists were generated using RawConverter (version 1.1.0.18, Scripps Research Institute). Protein identification was determined on the basis of at least three peptides with a minimum score of 30. False discovery rate (FDR) was calculated on the basis of a decoy search to a reversed protein sequence database and FDR was set to 1%.

To identify crosslinked peptides produced by the conjugation of thermolysin by A2ML1's thiol ester, an in-gel digest approach was also used. SDS-PAGE bands containing the protease conjugation product were prepared as described above, except that the gel bands were swelled with 0.1% v/v acetic acid, pH 3, and digested using pepsin instead of trypsin. Also, the samples were reduced using 10 mm DTT and alkylated with 30 mm of iodoacetamide prior to SDS-PAGE, to avoid variable cysteine propionamidylation. Digestion with pepsin was carried out overnight at 37°C. The digested peptides were extracted from the gel bands with ACN, and then purified using homemade C18 stage tips and analyzed by LC-MS/MS on an Orbitrap Eclipse Tribrid. The instrument performed HCD-fragmented MS2 scans in data-dependent acquisition mode, with a subsequent MS2 scan using electron transfer dissociation supplemented by HCD (EThcD) that was triggered by detection of the b2 and b3 product ions from the A2ML1 thiol ester peptide VQMPSGCGEQNMVL.

These MS2 spectra were analyzed using the Byonic search engine (version 3.7.13, Protein Metrics). The software was provided with the sequence of the pepsin-digested peptide covering A2ML1's thiol ester (VQMPSGCGEQNMVL) and the full protease sequence. Cysteine carbamidomethylation was used as a constant modification. Methionine oxidation and ester/amide crosslink formation between the thiol ester glutamine (Gln973) and lysine, serine, threonine, and tyrosine residues on the protease were included as variable modifications. Precursor and MS^2^ fragment ion mass tolerance were both set to 10 ppm. Protease specificity was set to FYLWAV, with four missed cleavages. FDR calculations or a threshold score were not used; MS^2^ assignments by Byonic were instead manually evaluated on the basis of coverage of both peptides participating in the crosslink and the known fragmentation pattern of the VQMPSGCGEQNMVL peptide.

### Assessing thermolysin inhibition with a casein-based activity assay

A total of 1.45 pmol of thermolysin or acetylated thermolysin were reacted with 0–16.3 pmol of WT or H1084N A2ML1 in 120 μl of 150 mm Tris-HCl, pH 7.8, 15 mm CaCl_2_ for 15 min at 37°C. Alternatively, reactions were performed at pH 5 with 1.45 pmol of thermolysin and 0–8.17 pmol A2ML1 in 120 μl of 50 mm sodium acetate, 100 mm NaCl, and 5 mm CaCl_2_ for 15 min at 37°C, after which the samples were neutralized by addition of 3 μl of 2 M Tris-HCl, pH 8.8. All samples were incubated with 40 μl of 4 mg/ml resorufin-labeled casein (Merck) for 60 min at 37°C before TCA was added to a final concentration of 2% to terminate the reactions and precipitate intact substrate. The precipitated substrate was removed by filtration using a MultiScreen Solvinert Filter plate (Millipore) by centrifugation at 500 g for 2 min. The filtrate was collected in a 96-well Nunclon Delta Surface plate (Thermo Fisher Scientific) and neutralized by addition of 2 M Tris-HCl, pH 8.8. The residual proteolytic activity was determined by measuring the absorbance at 574 nm using a FLUOstar Omega plate reader. Reactions were performed in triplicates.

### Depletion of collapsed A2M using LRP1-conjugated resin

A total of 200 mg of NHS-activated agarose (Pierce) and 600 μg of LRP1 (the A2M-binding cluster 1B, in fusion with a human Fc region) in 0.15 M TEAB, 0.15 M HEPES, pH 8.3 were mixed on a rotator at room temperature for 2 h. Following incubation, the resin was washed twice in HBS and the reaction was quenched with 50 mm Tris-HCl, pH 8 for 20 min, followed by a final washing step.

Approximately 0.6 mg of A2M N1088H were enriched in native A2M content by LRP1-based depletion of collapsed A2M. In each round of depletion, the LRP1-conjugated resin was mixed with A2M N1088H on a rotator for 30 min. The resin was then spun down and the supernatant was collected for future use. The resin was regenerated by eluting the bound collapsed A2M with 50 mm EDTA in HBS and washed twice in 10 mm Ca^2+^Cl_2_-supplemented HBS. Depletion was repeated until collapsed A2M was reduced to approximately 10% of the total protein amount.

## Data availability

The raw MS data files acquired during this study have been deposited to the ProteomeXchange Consortium via the PRIDE (31) partner repository with the data set identifier PXD020826, along with the corresponding Byonic search files and Skyline quantification files.

## Footnotes

3 S. L. Harwood, submitted for publication.
