# Correction: α2-Macroglobulin-like protein 1 can conjugate and inhibit proteases through their hydroxyl groups, because of an enhanced reactivity of its thiol ester

**DOI:** 10.1016/j.jbc.2020.100208

**Published:** 2021-02-10

**Authors:** Seandean Lykke Harwood, Nadia Sukusu Nielsen, Kathrine Tejlgård Jensen, Peter Kresten Nielsen, Ida B. Thøgersen, Jan J. Enghild

VOLUME 295 (2020) PAGES 16732–16742

The address for affiliation 2, Novo Nordisk is incorrectly attributed to General Research Technologies. It should be Global Research Technologies, Novo Nordisk A/S, Måløv, Denmark.

