# Supplementary material for: α2-Macroglobulin-like protein 1 can conjugate and inhibit proteases through their hydroxyl groups, because of an enhanced reactivity of its thiol ester
Source: J Biol Chem. 2021 Jan 13;295(49):16732–42. doi: 10.1074/jbc.RA120.015694 (PMC7864068; doi:10.1074/jbc.RA120.015694)
Supplement: Supplementary file 1 [file mmc1.docx]

**Supporting information**

**The thiol ester of A2ML1 has a C4B-like reactivity which enables the conjugation and inhibition of proteases through their hydroxyl groups.**

Seandean Lykke Harwood^1,2^, Nadia Sukusu Nielsen^1^, Kathrine Tejlgård Jensen^1^, Peter Kresten Nielsen^2^, Jan J. Enghild^1^

^1^Department of Molecular Biology and Genetics, Aarhus University, Aarhus 8000, Denmark

^2^General Research Technologies, Novo Nordisk A/S, Novo Nordisk Park, 2760 Måløv, Denmark.

**Contents of this document**

[Table S1. **Peptides covering the thiol ester glutamine**  2](#_Toc47969599)

[FIGURE S1. **MS2 spectra of the thiol ester-covering peptide from A2ML1**](#_Toc47969599) 3

[FIGURE S2. **Reducing SDS-PAGE of A2ML1 digested with HNE and CatG.** 4](#_Toc47969600)

[FIGURE S3. **Cross-linked peptide products** 5-8](#_Toc47969601)

[FIGURE S4. **SDS-PAGE of A2ML1’s auto-conjugation product** 9](#_Toc47969602)

[FIGURE S5**. Inhibition of thermolysin by A2ML1 at pH 5.** 10](#_Toc47969603)

[FIGURE S6. **MS2 spectra of the thiol ester-covering peptide from A2M**. 11](#_Toc47969604)

**Other supporting information:**

**MS data:** The raw mass spectrometry data files acquired during this study have been deposited to the ProteomeXchange Consortium via the PRIDE (33) partner repository with the dataset identifier PXD020826, along with the corresponding Byonic search files and Skyline quantification files.

Table S1. **Assignment of peptides covering the thiol ester glutamine from A2ML1 and A2M digested using pepsin.** These peptides were used as the basis for the PRM studies quantifying the reaction of the thiol ester with glycine or glycerol that are shown in Figure 1 and Figure 6. The spectra are shown in Figure S1 and Figure S6.

| Protein | Sequence | Modification | Theoretical  m/z | Observed  m/z | m/z  error (ppm) | z | Identification score  (Byonic, PEP2D) |
| --- | --- | --- | --- | --- | --- | --- | --- |
| A2ML1 | VQMPSGCGEQNMVL | Thiol ester  (-17.0266 Da)  at Q10 | 738.3210 | 738.3200 | -1.4 | 2 | 1.30E-05 |
| A2ML1 | VQMPSGCGEQNMVL | Glycine (+58.0056 Da)  at Q10 | 775.8360 | 775.8367 | 0.9 | 2 | 8.00E-06 |
| A2ML1 | VQMPSGCGEQNMVL | Glycerol (+75.0208 Da)  at Q10 | 784.3437 | 784.3438 | 0.1 | 2 | 1.30E-05 |
| A2M | LQMPYGCGEQN | Thiol ester  (-17.0266 Da)  at Q10 | 611.7470 | 611.7466 | -0.7 | 2 | 1.80E-07 |
| A2M | LQMPYGCGEQN | Glycine (+58.0056 Da)  at Q10 | 649.2630 | 649.2623 | -1.1 | 2 | 2.20E-06 |
| A2M | LQMPYGCGEQN | Glycerol (+75.0208 Da)  at Q10 | 657.7707 | 657.7705 | -0.3 | 2 | 5.40E-05 |


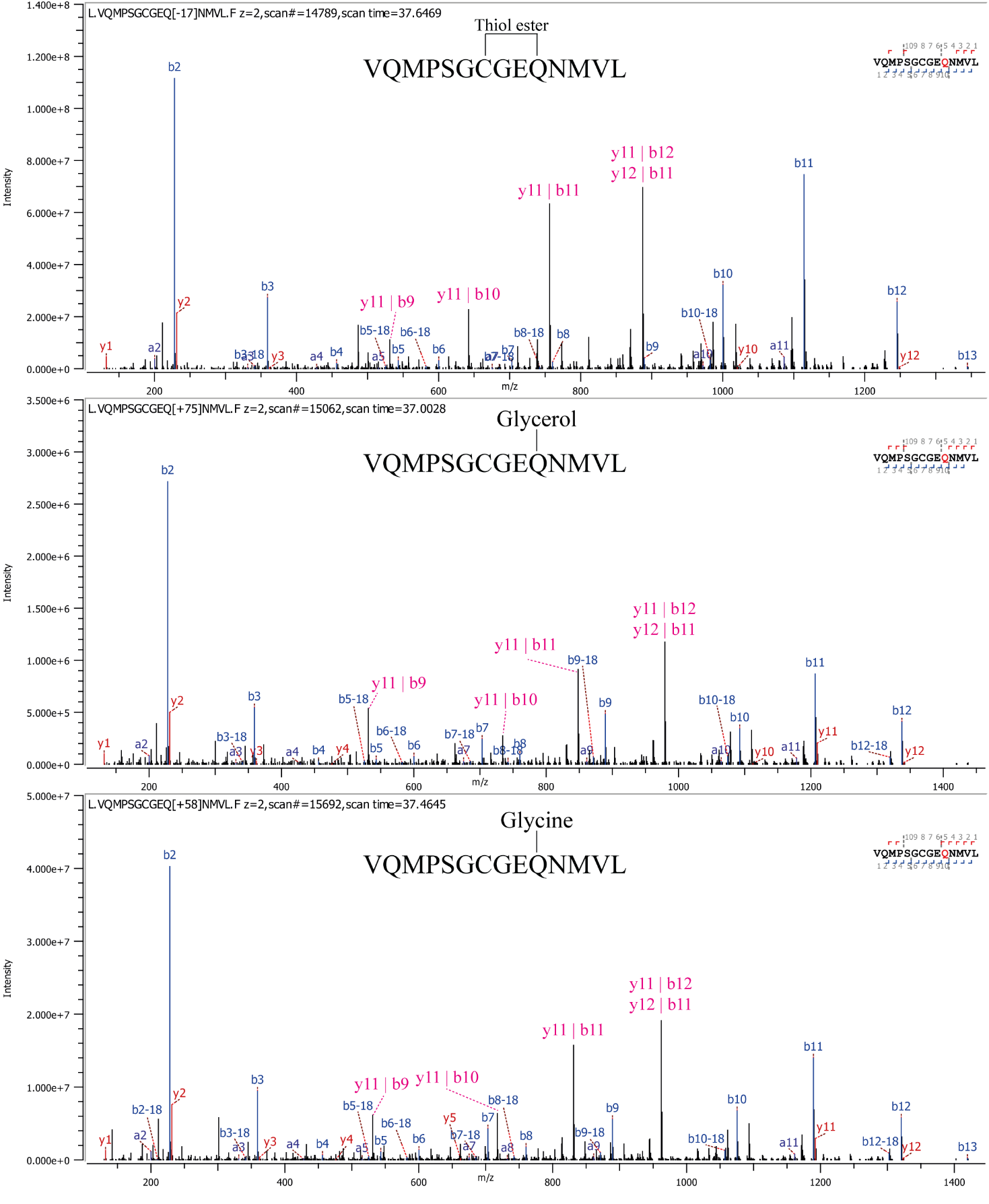
FIGURE S1. **MS2 spectra of the thiol ester-covering peptide from A2ML1**. HCD-fragmented MS2 spectra for the thiol ester-covering peptide, modified either by an intact thiol ester or conjugation of the thiol ester glutamine to glycine or glycerol, are shown. y- and b-type product ions identified by the Byonic search engine are labelled in red and blue, respectively, whereas product ions produced by multiple fragmentation events were manually identified and are labelled in magenta.


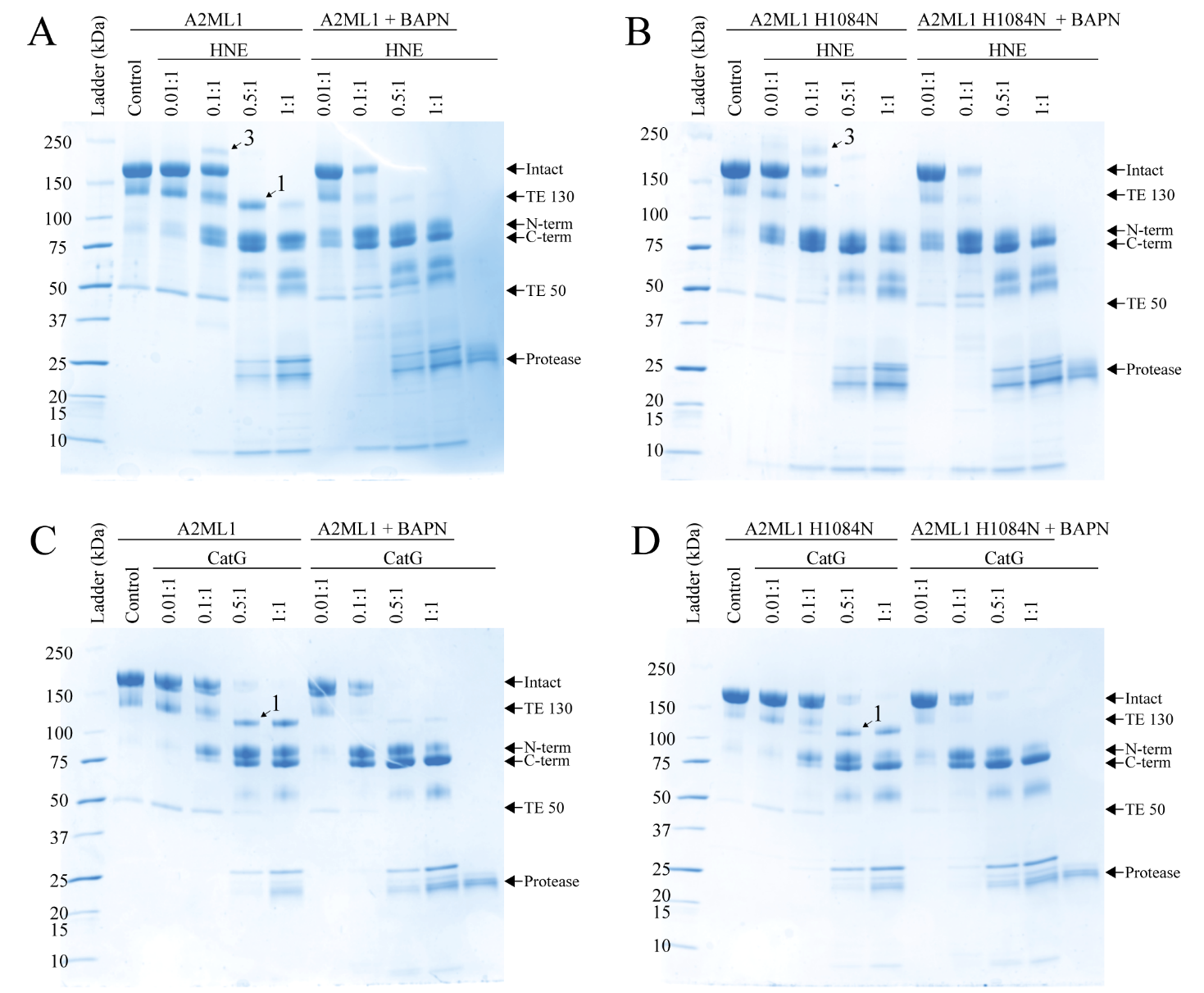


FIGURE S2. **Reducing SDS-PAGE of A2ML1 digested with human neutrophil elastase and cathepsin G.** Wildtype (**A** and **C**) or H1084N (**B** and **D**) A2ML1 were digested with HNE (**A** and **B**) and CatG (**C** and **D**) at various molar ratios of protease:A2ML1 as indicated for 15 min at 37 °C, after which thermolysin was inhibited with 2 mM PMSF. Digestions were also performed with 50 mM BAPN present. Both A2ML1s are initially cleaved in their bait regions by HNE and CatG, with secondary cleavage occurring at higher protease ratios. Both A2ML1s form a ~120 kDa protease conjugation product (band “1”) with CatG, which has both amine and hydroxyl groups, but only wildtype A2ML1 form a ~120 kDa protease conjugation product with HNE, which has hydroxyl groups but not amine groups. The intra-A2ML1 conjugation product is labelled as band “3”. Band “1” was confirmed by Edman sequencing and in-gel digestion followed by LC-MS/MS to contain the C-terminal fragment of A2ML1, as well as either HNE or CatG.


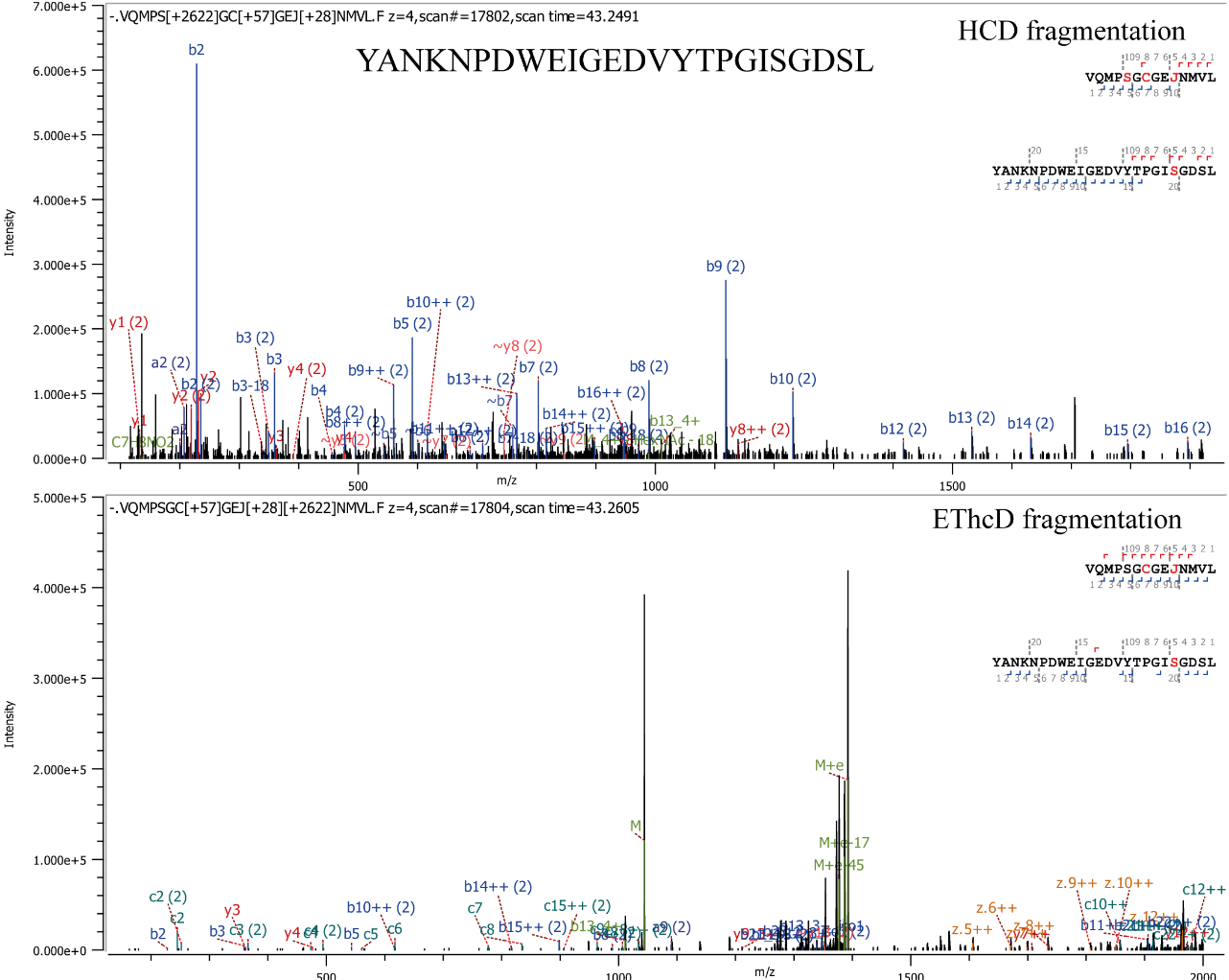

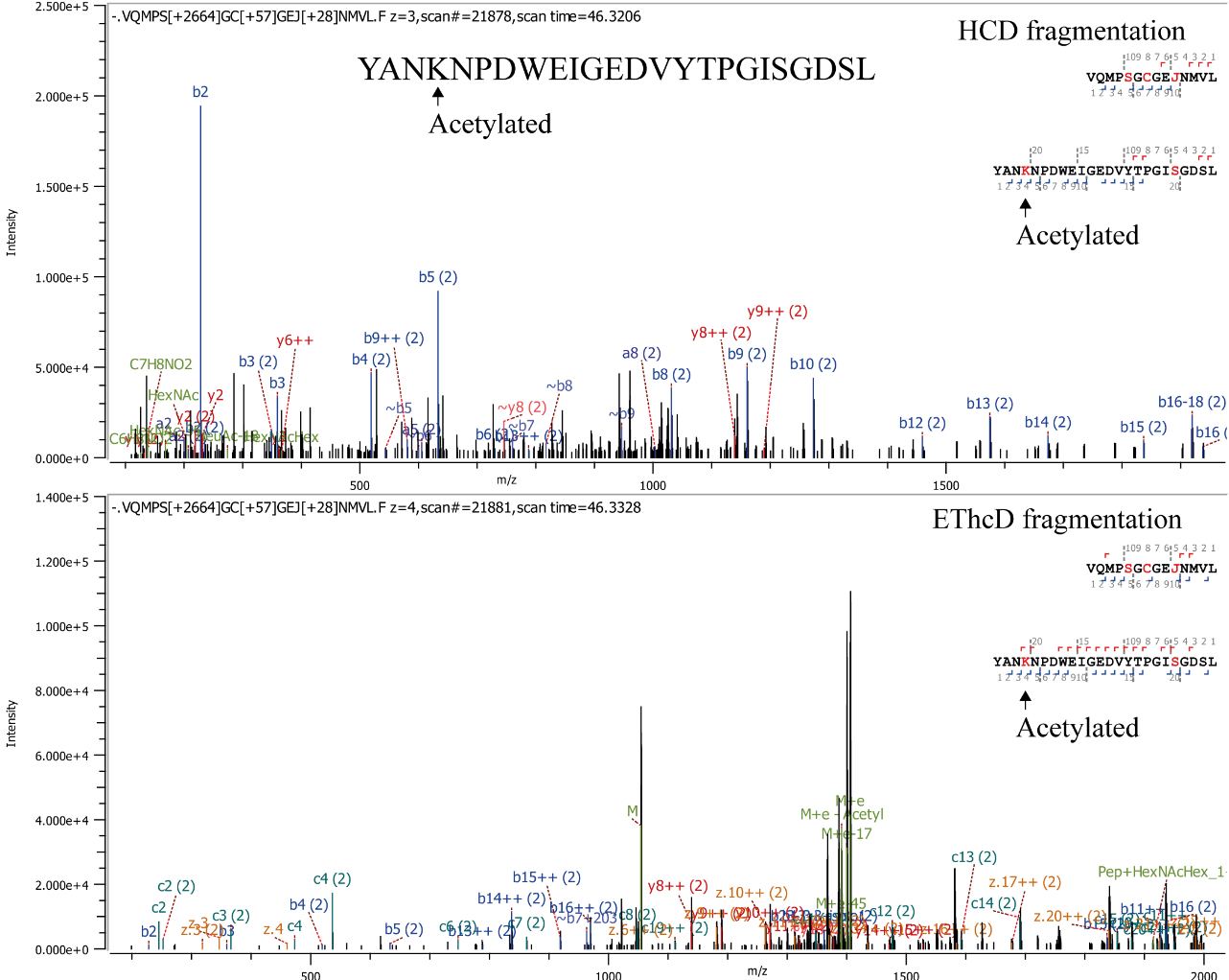


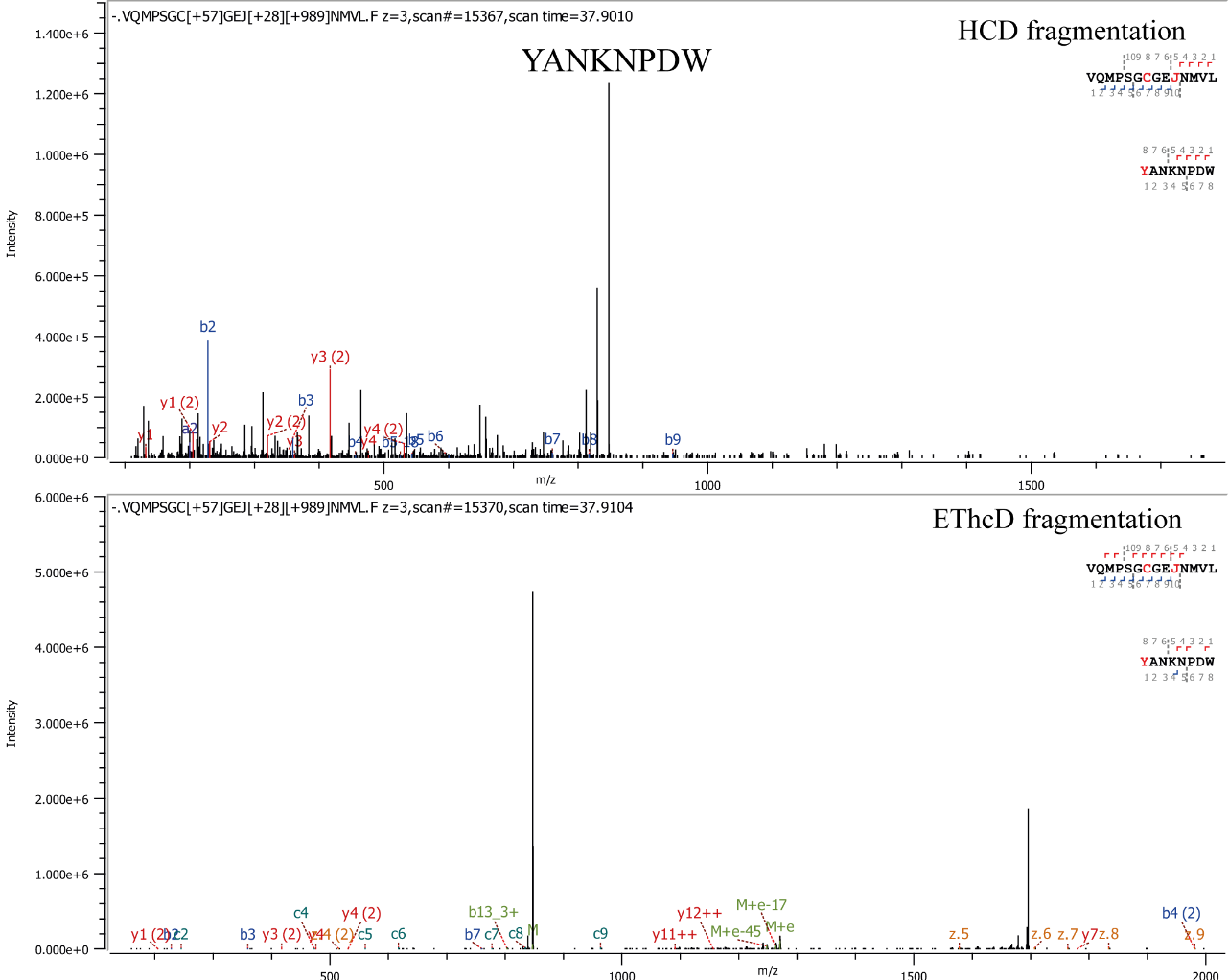

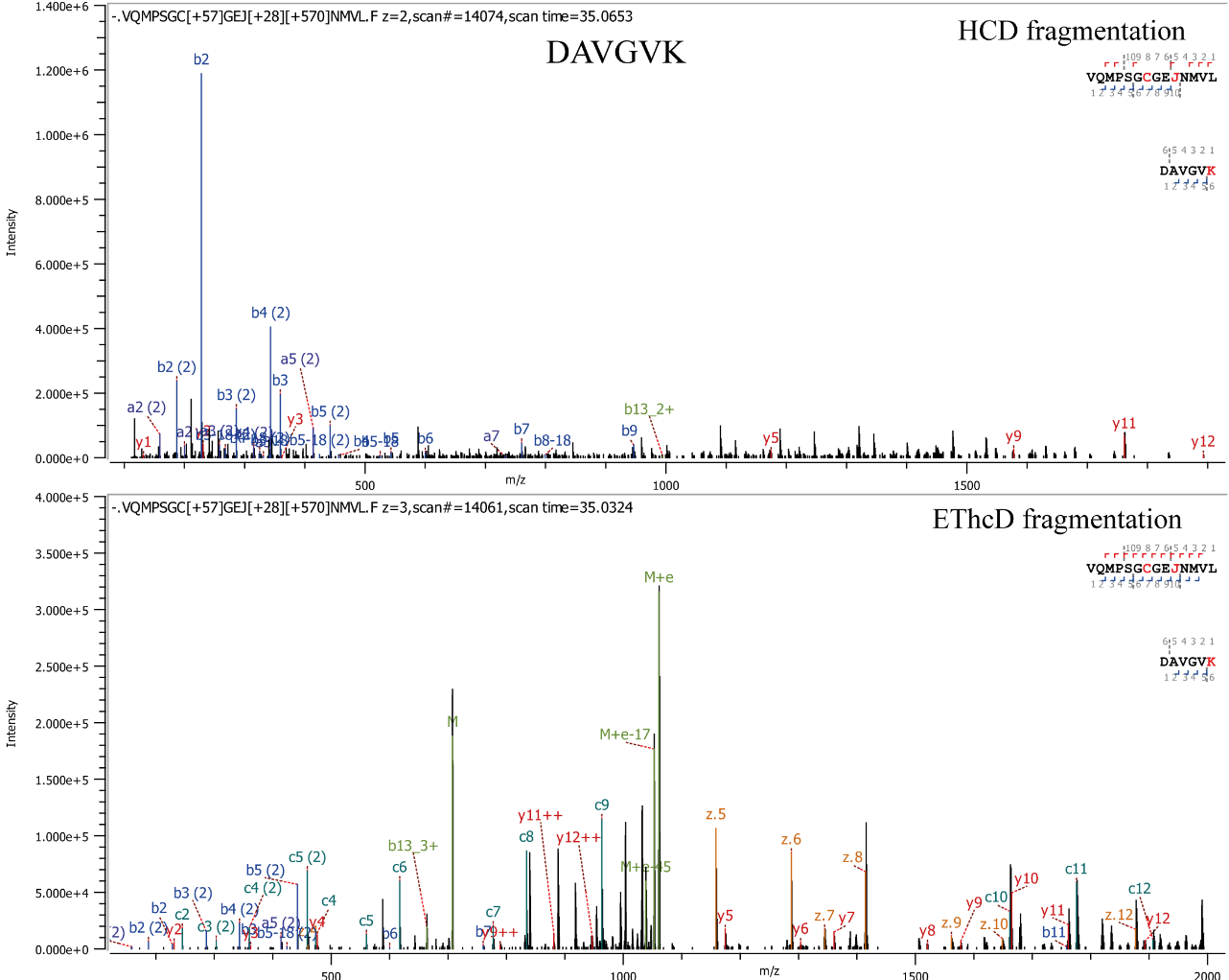

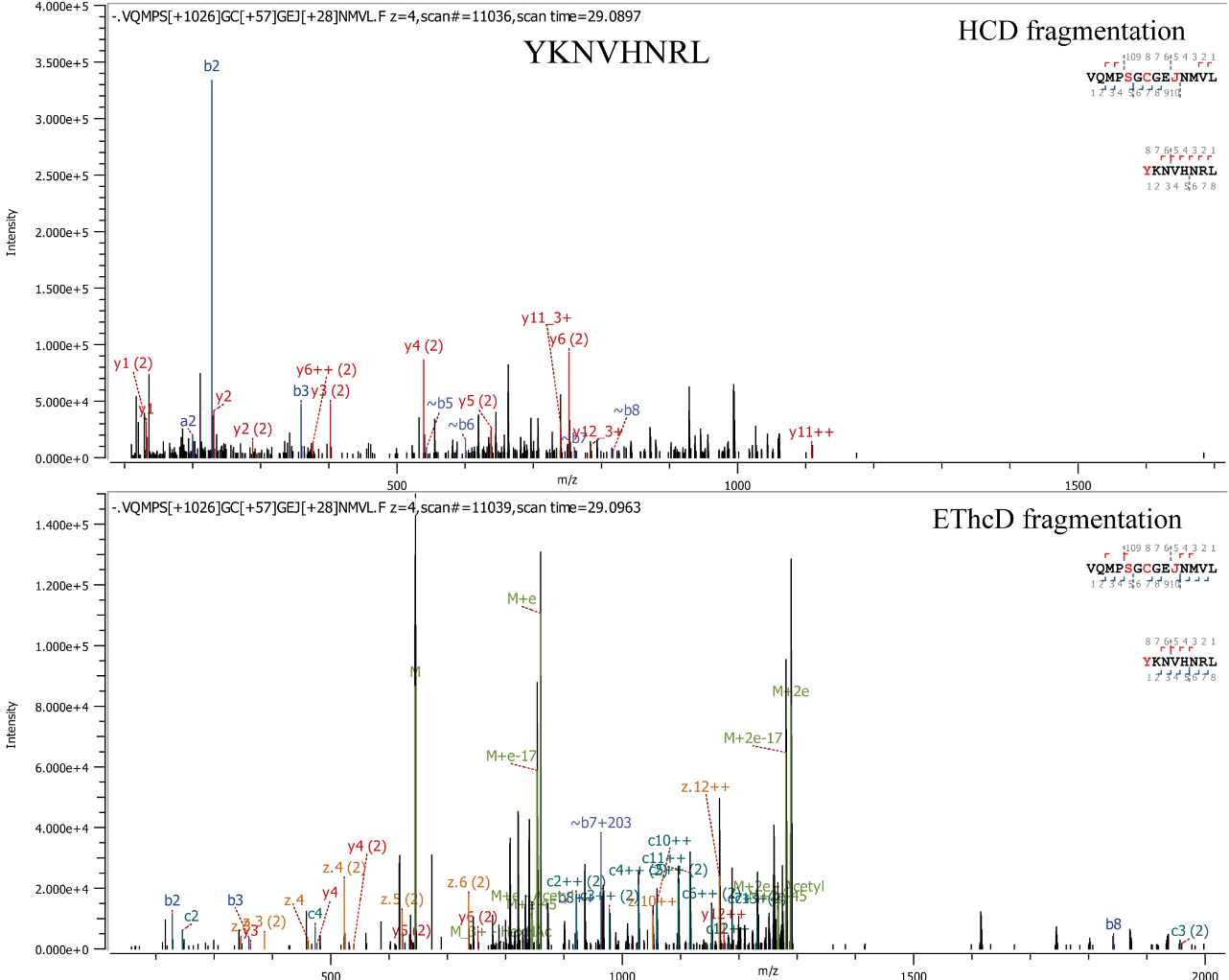

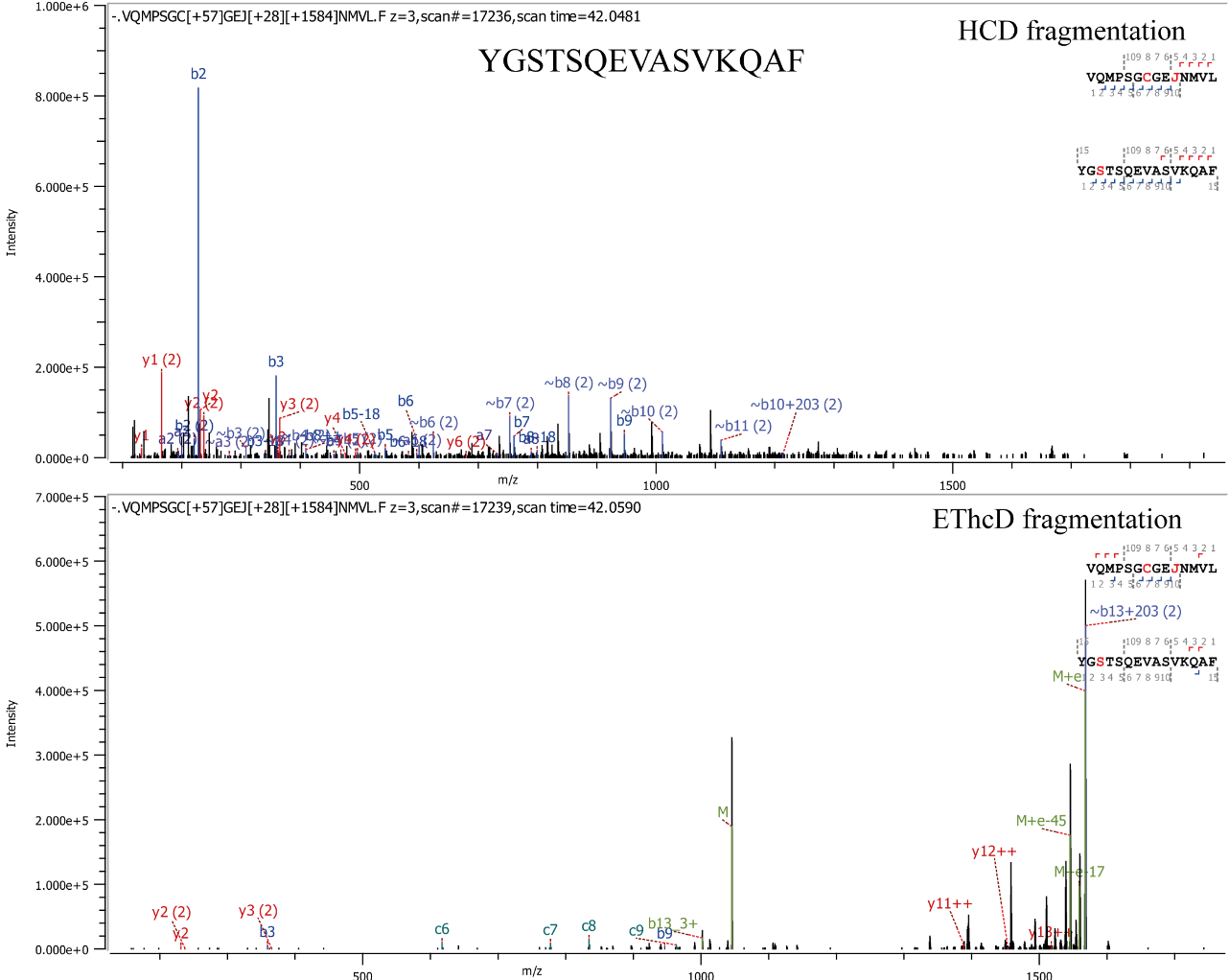

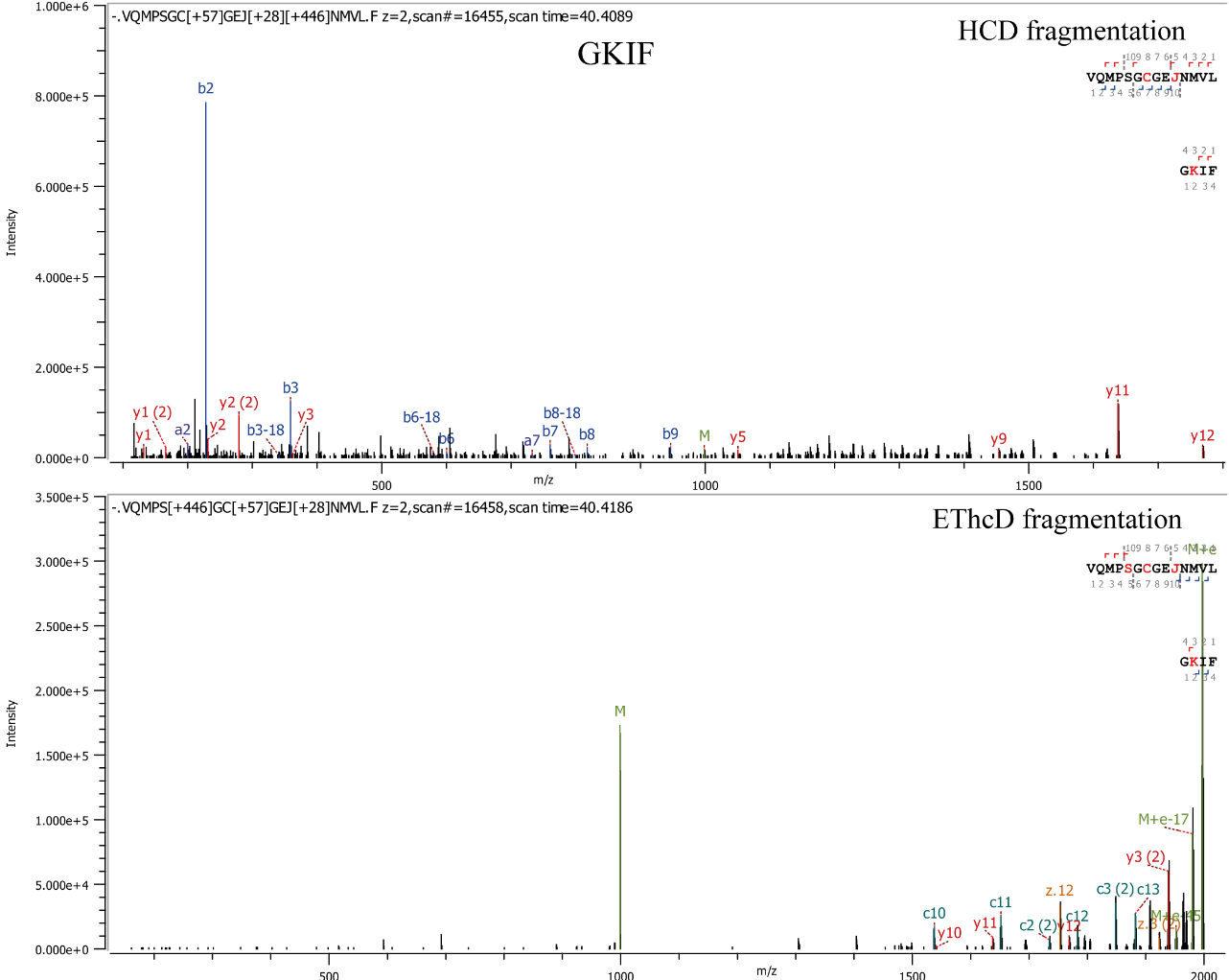


FIGURE S3. **Cross-linked peptide products from thiol ester conjugation to thermolysin.** Unmodified or acetylated thermolysin was used to digest wildtype A2ML1, and the ~150 kDa conjugation product seen in SDS-PAGE was digested by pepsin and analyzed by LC-MS/MS. Peptides were first fragmented using higher-energy collision dissociation (HCD); if the intense b2 and b3 product ions of the thiol ester-covering peptide VQMPSGCGEQNMVL were detected (see Figure S1), the peptide was then fragmented using electron transfer dissociation supplemented with HCD (EThcD). The data were analyzed using the Byonic search program; note that the thiol ester glutamine residue was defined as a unique “J” residue for the sake of avoiding cross-link consideration involving other non-thiol ester glutamine residues. This identified 6 cross-links to unmodified thermolysin and 1 cross-link to acetylated thermolysin with sufficient coverage of both participant peptides in one or both fragmentation methods for a reasonably confident assignment. However, the cross-link position on thermolysin was only determined for the peptides YANKNPDWEIGEDVYTPGISGDSL, which was identified in both the unmodified and acetylated thermolysin sample, and for DAVGVK and GKIF. The identification of YANKNPDWEIGEDVYTPGISGDSL participating in the cross-linked peptide in the acetylated sample allowed the K4 residue to be ruled out as the conjugation site due to its modification by acetylation. In the other 3 peptides, fragmentation coverage did not sufficiently restrict the conjugation site to a sequence stretch containing a single nucleophilic residue. The difficulty of correctly identifying the conjugation site is illustrated by the fact that the conjugation position on the thiol ester peptide was erroneously identified by the Byonic search engine as the S5 residue instead of Q10 for two cross-linked peptides.


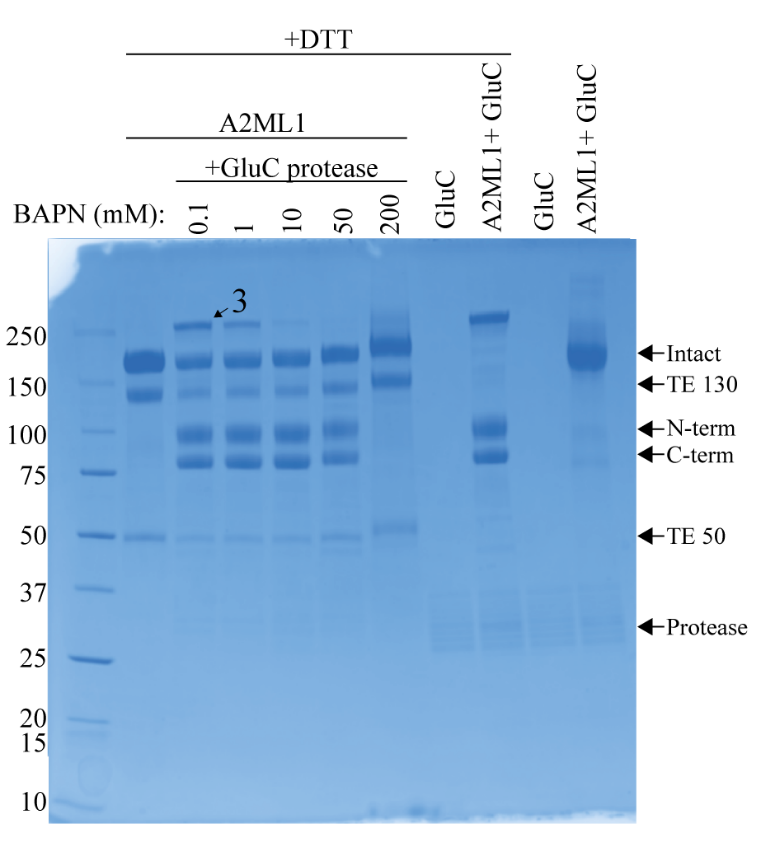


FIGURE S4. **SDS-PAGE of A2ML1’s auto-conjugation product.** Wildtype A2ML1 was cleaved using a 1:10 molar ratio of GluC:A2ML1 with a titration series of BAPN and analyzed by reducing SDS-PAGE. Some A2ML1 was also cleaved using a 1:1 molar ratio of GluC:A2ML1 without BAPN and analyzed by both reducing and non-reducing SDS-PAGE. Band “3,” which was determined by MS and Edman degradation to contain both the N- and C-terminal fragment of bait region-cleaved A2ML1, is dependent on thiol ester-mediated conjugation, as seen by its disappearance at 10-200 mM BAPN. This thiol ester-mediated conjugation does not lead to A2ML1 dimerization under non-reducing conditions and therefore takes place within a single A2ML1 protein. Band “3” is therefore an intra-A2ML1 conjugation product. In-gel digestion and LC-MS/MS, as well as Edman sequencing, showed that band “3” contains both the N- and C-terminal bait region fragments of A2ML1, but not the GluC protease. Cross-linked peptides determining the conjugation site were not successfully identified.


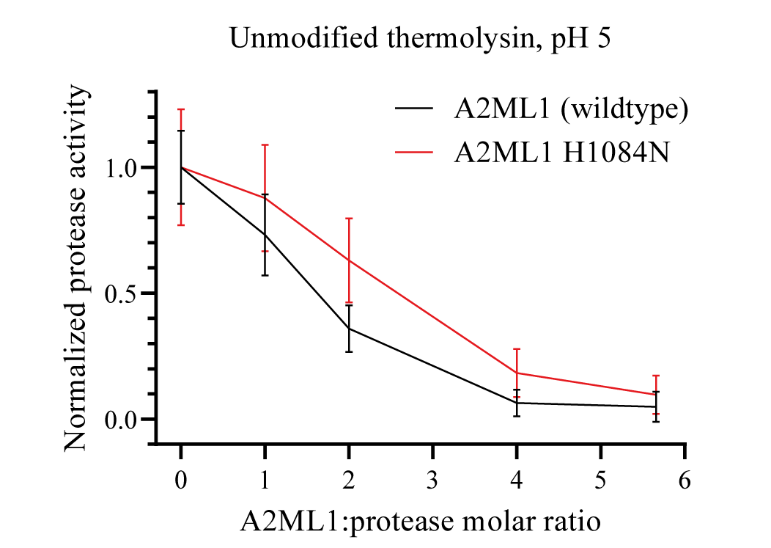


FIGURE S5**. Inhibition of thermolysin by A2ML1 at pH 5.** Unmodified thermolysin and wildtype or H1084N A2ML1 were incubated for 1 hour at 37°C, after which the samples were neutralized with Tris. The residual proteolytic activity was determined as described in figure 4. The inhibition of thermolysin at pH 5 by either A2ML1 is similar, indicating that the hydroxyl thiol ester reactivity of wildtype A2ML1 does not improve protease inhibition at pH 5. Roughly 4 moles of A2ML1 are required to inhibit 1 mol of thermolysin at pH 5, in contrast to the 2 moles of A2ML1 which were required at pH 7.8 as shown in Figure 4.


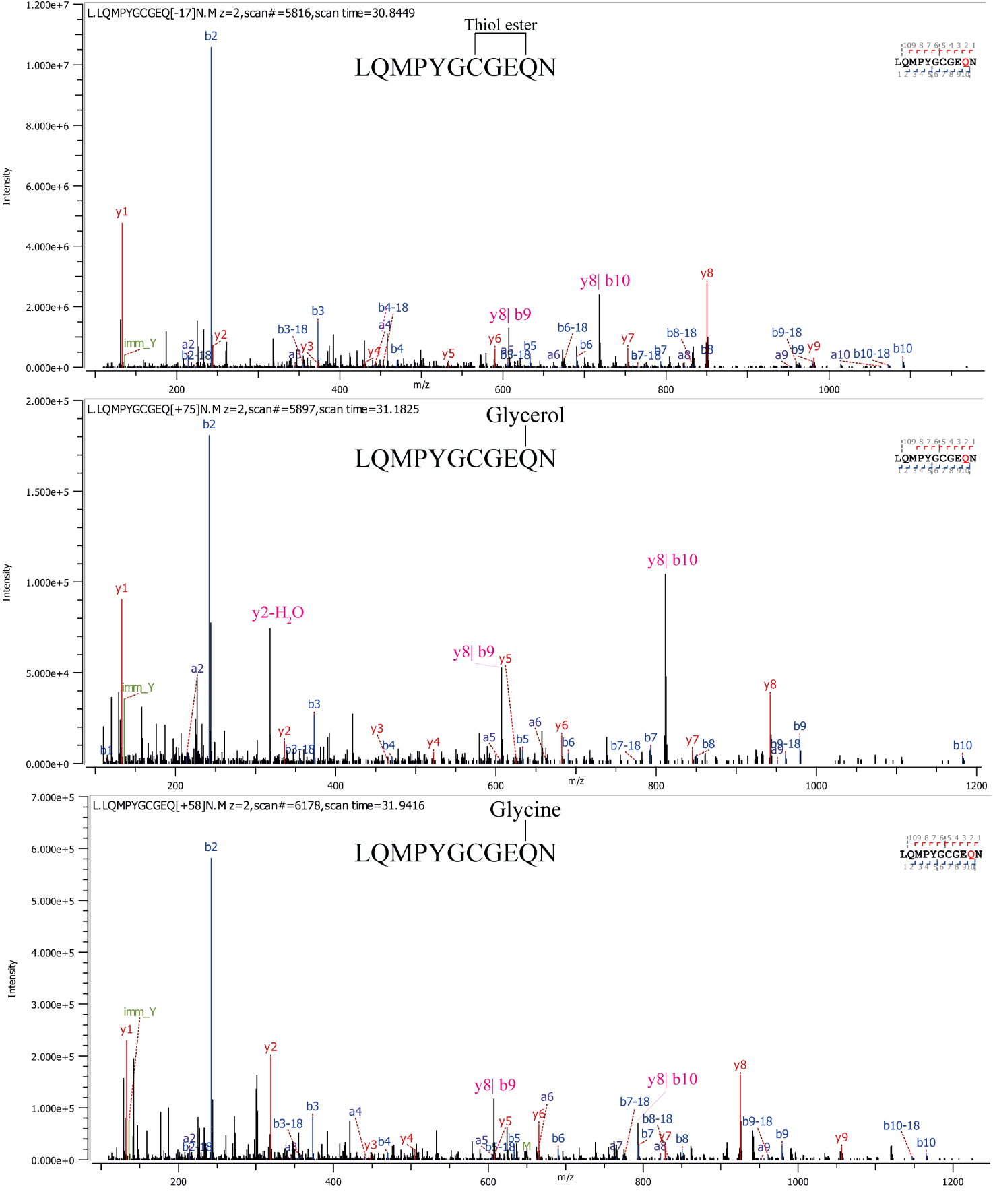


FIGURE S6. **MS2 spectra of the thiol ester-covering peptide from A2M**. HCD-fragmented MS2 spectra for the thiol ester-covering peptide, modified either by an intact thiol ester or conjugation of the thiol ester glutamine to glycine or glycerol, are shown. y- and b-type product ions identified by the Byonic search engine are labelled in red and blue, respectively, whereas manually assigned products of double fragmentation or fragmentation and neutral losses are shown in magenta.­
